# Molecular tracking of interactions between progenitor and endothelial cells via Raman and FTIR spectroscopy imaging: a proof of concept of a new analytical strategy for in vitro research

**DOI:** 10.1007/s00018-023-04986-3

**Published:** 2023-10-18

**Authors:** Karolina Augustyniak, Aleksandra Pragnaca, Monika Lesniak, Marta Halasa, Agata Borkowska, Ewa Pieta, Wojciech M. Kwiatek, Claudine Kieda, Robert Zdanowski, Kamilla Malek

**Affiliations:** 1 Department of Chemical Physics, Faculty of Chemistry, Jagiellonian University in Krakow, Gronostajowa 2, 30-387 Krakow, Poland; 2 Doctoral School of Exact and Natural Sciences, Jagiellonian University in Krakow, Prof. S. Lojasiewicza 11, 30-348 Krakow, Poland; 3 Laboratory of Molecular Oncology and Innovative Therapies, Military Institute of Medicine-National Research Institute, Szaserow 128, 04-141 Warsaw, Poland; 4 Transplant Immunology, The Houston Methodist Research Institute, Houston, TX, USA; 5 Postgraduate School of Molecular Medicine, Medical University of Warsaw, Zwirki i Wigury 61, 02-091 Warsaw, Poland; 6 Institute of Nuclear Physics, Polish Academy of Sciences, Radzikowskiego 152, 31-342 Krakow, Poland; 7 Center for Molecular Biophysics, UPR4301 CNRS, Orleans, France; 8 Department of Surgery, The Houston Methodist Hospital, Houston, TX, USA

**Keywords:** Mouse aorta gonad mesonephros endothelial cells 11.5 (MAgEC11.5), Mouse brain microvascular endothelial cells (MBrMEC), Intercellular interactions, Raman and FTIR imaging, Multivariate analysis

## Abstract

Circulating endothelial cell progenitors originating from the bone marrow are considered to be a powerful tool in the repair of endothelium damage. Due to their unique properties, endothelial progenitors are now broadly investigated to assess their clinical significance in diseases e.g., associated with brain endothelial dysfunction. However, their distinction in terms of the expression of specific markers remains ambiguous. Additionally, endothelial progenitor cells may change their repertoire of markers depending on the microenvironment of the tissue in which they are currently located. Here, we applied the label-free Raman and FTIR imaging to discriminate mice brain endothelium and endothelial progenitors. Cells cultured separately showed distinctly different spectral signatures extracted from the whole cellular interior as well as the detected intracellular compartments (nucleus, cytoplasm, perinuclear area, and lipid droplets). Then, we used these spectroscopic signals to examine the cells co-cultured for 24 h. Principal cluster analysis showed their grouping with the progenitor cells and segregation from brain endothelium at a level of the entire cell machinery (in FTIR images) which resulted from biochemical alternations in the cytoplasm and lipid droplets (in Raman images). The models included in partial least square regression indicated that lipid droplets are the key element for the classification of endothelial progenitor-brain endothelial cells interactions.

**Supplementary Information:**

The online version contains supplementary material available at 10.1007/s00018-023-04986-3.

## Introduction

Circulating endothelial progenitor cells (EPCs) originating from the bone marrow are considered to be a powerful tool in the repair of endothelium damage [1]. Although there are some controversies related to EPCs classification due to their specific markers expression, two populations can be defined as early and late EPCs [2]. Both are critical for endothelium repair; nonetheless, they use different mechanisms for this activity. Early EPCs have low proliferative potential and secret a set of proangiogenic cytokines, whereas the proliferative potential of late EPCs is high. The late EPCs’ recruitment (via endogenous tissue ischemia and mediated cytokines) and the differentiation into mature endothelial cells by adaptation to local environment promote vasculogenesis and angiogenesis [3]. Due to their unique properties, EPCs have been broadly investigated to assess their clinical significance in diseases associated with endothelial dysfunction as occurring in the brain ischemic stroke [4]. EPCs systemically delivered into mice protected against cerebral ischemic injury and enhanced long-term neurobehavioral outcomes [5]. However, to this day, clinical trials based on EPCs transplantation have not provided successful outcomes [6] or are still ongoing [7]. Our research showed recently that EPCs injected into mice could migrate into the brain and play as successful providers of the gene construct to destroy the proteins involved in Alzheimer’s disease (AD) [8]. EPC dysregulation has been observed in patients suffering from AD showing contradictory outcomes. On the one hand, AD patients presented with decreased EPCs function compared to healthy control suggesting their role in AD development [1]. On the other hand, other data showed no differences in EPCs levels compared with healthy control, excluding EPCs as potential AD markers [2]. Finally, AD patients with moderate to severe dementia showed significantly increased levels of circulating progenitor cells compared to healthy elderly control [3]. Such results partly suggest a need for the development of more sensitive diagnostic techniques. To sum up, EPCs seem to be a promising therapeutic tool that has already been nicely revised here [9, 10]. However, the repairment role of EPCs in e.g., neurodegenerative diseases, among others, is still poorly understood due to their plasticity and adaptation to the microenvironment once locally recruited. For more comprehensive diagnostics allowing a better recognition of EPCs from other cells, new solutions are highly in demand.

Although many functions of EPCs have been well understood, including proliferative potential and capacity to differentiate into mature endothelial cells, their adaptability makes their distinction in terms of expression of specific markers ambiguous. For this reason, highly-advanced spectroscopic techniques have gained importance as they allow to obtain comprehensive data associated with the biochemical composition of cells [11]. In our study, we used Raman (RS) and Infrared (FTIR) spectroscopies to investigate the possibility of distinguishing the endothelial progenitor (MagEC11.5 [12, 13]) from brain endothelium cells (MBrMEC [14, 15]) and further track their intercellular interactions. FTIR and Raman (RS) spectroscopies are complementary methods that demonstrate information about principal biomolecules, such as lipids, proteins, nucleic acids, and carbohydrates. RS is sensitive to lipids, aromatic amino acids, nucleotides, and hemoproteins, while FTIR is more specific for secondary structures of proteins, esterified lipids, nucleic acids, and carbohydrates [16]. Both methods are sensitive, label-free, and non-destructive. Their combination enables the identification of the overall biochemical composition of bio-samples at the microscopic scale since both signal readouts can be provided by the conjunction of a spectrometer with a microscope. FTIR spectroscopy imaging allows the measurement of a large sample area in a few minutes. The focal plane array detector (FPA) is built of a 128x128 elements matrix (5.5 μm x 5.5 μm pixels) and acquires spectra from an area of ca. 700 μm x 700 μm in a single shot [17]. It gives a lateral resolution of 7.6 μm at 2500 cm^−1^. Thus, one obtains an average signal from the whole cell [18]. In turn, RS spectroscopy imaging scans a cell with max. lateral resolution of ca. 0.3 μm and shows the spatial distribution and chemical composition of cellular compartments.

The implementation of FTIR and Raman imaging to analyze biological objects such as cells or tissues is an approach that has been used in research for many years and showed numerous applications. For example, a high Raman scattering cross section of lipids is a powerful tool for tracking the formation of lipid droplets (LDs) and determining their degree of unsaturation, which was proposed as a marker of inflamed endothelium [19, 20]. In turn, FTIR imaging revealed its cellular metabolism showing a decrease in lipid level with a simultaneous increase of carbohydrates [17]. Moreover, FTIR spectroscopy is an excellent technique to observe structural changes in secondary conformations of proteins [21, 22], carbohydrates [23, 24], and DNA [25]. Furthermore, both techniques have been successfully applied by us for the differentiation of endothelial cell lines, such as HMEC-1, EA.hy926, and HAoEC [18]. An in-depth Raman spectral analysis revealed subtle line-specific differences. Nuclei of HMEC-1 and HAoEC cells showed a higher DNA/RNA ratio than in the EA.hy926 cell line, whereas nucleoli identified by the Raman RNA signal were not detected in HAoEC cells. Furthermore, the highest number of lipid droplets was observed in HMEC-1, but the total content of cytoplasmic lipids was extremely high in the EA.hy926 cell line as FTIR data indicated [18]. Raman microscopy also supported the identification of murine primary endothelial cells isolated from various organs by showing that endothelium has a unique spectroscopic signature that can be discriminated from the spectrum of an organ [26]. Recently, a Raman study reported a high potential of this technique to recognize the effect of the Epstein-Barr virus on brain microvascular endothelial cells by the alternation of the spectral markers of cholesterol, polysaccharides, and nucleic acids [27]. Nowadays, RS and FTIR spectroscopies have been increasingly used to distinguish the differentiation levels of various mouse and human cells from blood [28], neural system [29, 30], and kidneys [31]. These results reveal spectral differences between cells but also allow the construction of prediction models with high specificity and sensitivity [28, 30, 32].

Therefore, this work aimed to assess the ability of molecular spectroscopy imaging to recognize the progenitor cells from brain endothelium and further track their interaction pathway. We determined first spectral features of individual cell lines at various levels of cellular organization and next, we observed their changes due to interactions in 2-dimensional co-culturing. Finally, we concluded that microvascular brain endothelium adopts molecular characteristics of the progenitor cells and developed a prediction model to assess the degree of this cellular transformation. Our study can pave the pathway to studying the biology of EPCs and facilitate research toward their use in cell therapy. According to our best knowledge, no reports have proposed such a non-label approach for tracking cellular interactions, and this paper is the first attempt to use both techniques for this application.

## Materials and methods

### Cell culturing

Two primary cell lines were used in the experiment, i.e., Mouse Aorta Gonad Mesonephros Endothelial Cells 11.5 (referred to as MAgEC11.5) and Mouse Brain Microvascular Endothelial Cells (referred to as MBrMEC) immortalized as described elsewhere [12, 14]. To confirm the progenitor character, the MAgEC11.5 were previously characterized due to the expression of specific markers [12, 13]. Both cell lines were cultured in OPTI-MEM (Gibco, UK) supplemented with 2% FBS (Gibco, UK). The cells were passaged every 3-4 days, and they were regularly tested against mycoplasma. Mycoplasma-free cells were maintained at 37°C in a humidified atmosphere with 5% CO_2_. Cells between the 5th and the 9th passages were used for all experiments. MAgEC11.5 and MBrMEC cells were seeded in a 6-well plate with sterile 25 mm x 2 mm CaF_2_ windows (Crystran, UK). The experiment consisted of a culture of single cell lines (4x10^5^/well) and a co-culture of both cell lines (2x10^5^/well for each line) incubated for 24 and 4 h. The latter (cell adhesion only) was the negative control. After the incubation (37°C, 5% CO_2_), cells were washed twice with PBS (Corning, USA) and fixed with 2.5% glutaraldehyde (SERVA, Germany). The fixed cells were washed three times with PBS and stored at 4°C until imaging for less than 3 days. Two replicates were prepared.

### Fluorescence microscopy

MAgEC11.5 and MBrMEC cells were seeded on a 4-well Lab Tek II Chamber slide (ThermoFisher Scientific, USA) coated with 0.1% gelatin (Sigma Aldrich, Germany) dissolved in PBS at 4x10^5^/well. Cells were cultured for 24 h in OPTI-MEM (Gibco, UK) supplemented with 2% FBS (Gibco, UK) at 37°C, 5% CO_2_. After 24 h, intracellular lipids were stained with the use of a Vybrant Multicolor Cell Labeling kit (Invitrogen, UK). In that protocol, cells were incubated for 1 h at 37°C, 5% CO_2_ with 1 μM/mL DIL (Abs 549) dissolved in FBS free medium. Afterward, cells were washed with PBS (Corning, USA) 3 times for 10 min and then fixed with 1.5% glutaraldehyde (SERVA, Germany) for 1 h at 4°C. The fixed cells were washed with PBS twice and incubated for 1 h at RT in the dark with Phalloidin-Atto 488 (Sigma-Aldrich, Germany) diluted with PBS in 1:100 concentration to stain the cytoskeleton. Stained cells were washed with PBS twice. Nuclei were stained with bisBenzimide H33342 trihydrochloride (Sigma, Germany) diluted 1:1000 with PBS. Incubation proceeded at RT in the dark for 15 min, followed by washing with PBS (2 times). Stained slides were sealed using Vectra Shield Vibrance – Antifade Mounting Medium with DAPI (Vector Laboratories, USA). Images were acquired using a Zeiss Axio Observer microscope equipped with an Axiocam 530 mono camera (Zeiss, Germany). CA ZEN Blue Edition software (ver. 3.4, Zeiss, Germany) was used for the analysis.

### Raman spectroscopy imaging

Raman imaging was carried out with the use of a WITec confocal Raman imaging system (WITec Alpha 300R Raman microscope, WITec, Germany). Raman spectra were acquired with an excitation laser at 532 nm (power of 23 mW), which was coupled to the microscope via an optical fiber with a core diameter of 50 µm. The microscope was equipped with a CCD detector cooled to −80°C. Cells immersed in PBS solution and mounted on a CaF_2_ window were illuminated through a 60× water objective (NA: 1.0, Zeiss). Raman images were recorded with a step size of 1 μm. High-Resolution Raman images were acquired with a step size of 0.3 μm giving a lateral resolution of 0.32 μm. Raman spectra were collected with an integration time of 0.5 s and a spectral resolution of 3 cm^−1^. For each experimental group, ca. 40 images were recorded.

### FTIR spectroscopy imaging

FTIR images were collected using a Hyperion 3000 FTIR microscope (Bruker Optics, Ettlingen, Germany) and an Agilent 670‐IR FTIR spectrometer connected with a 620‐IR microscope (Santa Clara, California, USA).

A focal plane array (FPA) detector cooled with liquid nitrogen was coupled with the microscopes. The used detectors consist of a matrix of 4096 (64 × 64 grid format) and 16 384 pixels (128 × 128 grid format), respectively. IR images were acquired in transmission mode. Samples were illuminated through 15 × objective and condenser optics with NA of 0.62 and a projected FPA pixel size of 5.5 μm × 5.5 μm. FTIR spectra of cells were acquired by co‐adding 128 scans in the region of 900–3700 cm^−1^ with a spectral resolution of 4 cm^−1^. 40 images were collected for each experimental group.

### Preprocessing of Raman and FTIR images

Raman images were preprocessed using a cosmic ray removal filter with a size of 3 and a dynamic factor of 8. For baseline correction, the 3rd-grade polynomial was used. Next, chemical maps were constructed based on integral intensity in spectral regions specific for organic matter (2800–3030 cm^−1^), lipids (2830–2900 cm^−1^), and nucleic acids (790–810 cm^−1^). Afterward, k-means cluster analysis (KMCA) with a Manhattan distance method and randomized k-means distribution was performed to segregate the Raman image of a single cell into classes attributed to cytoplasm, nucleus, perinuclear area, and lipid droplets (WITec 5.0 software, Germany). The realignment of clusters was finished once the obtained cluster maps fully correlated with chemical images and spectral profiles of classes revealed characteristic RS bands. Raman spectra extracted for KMC classes were next truncated in the spectral region of 500–3050 cm^−1^, baseline-corrected (10 iterations), and smoothed according to a Savitzky–Golay protocol (11 points) using an OPUS ver. 7.0 software (Ettlingen, Germany).

Pre-processing of FTIR images was performed using CytoSpec (ver. 2.00.01) [33] and MatLab (R2017a, Natick, Massachusetts) software. Subsequently, water vapor removal, PCA-based denoising (20 PCs), and smoothing with a Savitzky-Golay algorithm (25 points) were performed on collected images (CytoSpec, MatLab). Based on the distribution maps of proteins (1620-1680 cm^−1^), single cells with the highest signal-to-noise ratio were chosen. Spectra from selected regions of interest (ROIs) were further averaged and extracted. As a result, each cell was represented by a single FTIR spectrum. Resonant Mie Scattering correction (EMSC) using seven principal components was performed on extracted FTIR spectra (MatLab) [34].

### Chemometric analysis

Chemometric analysis was performed using Unscrambler X 10.3 software (CAMO Software AS., Norway). Before analysis, Raman spectra were smoothed (Savitzky–Golay, 3rd-order polynomial, 15 pts) while FTIR spectra were transformed into a second derivative (Savitzky–Golay, 2nd-order polynomial, 13 pts). In the next step, all spectra were baseline-corrected (offset) and vector normalized. Such pre-processed data sets were used for unsupervised principal component analysis (PCA). PCA was performed in the bio-regions of 1000-3050 and 500-3050 cm^−1^, for mean-centered FTIR and Raman spectra, respectively, with an SVD algorithm of cross-validation and 7 principal components. As a result of PCA, score plots and loadings graphs were generated to show grouping and variance within Raman and FTIR spectra of cells cultured separately and together in one batch.

Partial least square regression (PLSR) was performed on the same data sets as for PCA. Ca. 80% of the spectra of the whole single cells and their cellular compartments from separately cultured cells were used to calibrate and validate models using a NIPALS algorithm with full cross-validation and 10 factors. Then the models were tested on the remaining data set. Next, these models were employed to classify co-cultured MAgEC11.5 and MBrMEC cells.

All graphs presenting spectra and the results of the chemometric analysis were prepared using Origin 2021 software.

## Results

### Morphology of endothelial progenitor MAgEC11.5 and brain endothelial MBrMEC cells

**Fig. 1:**
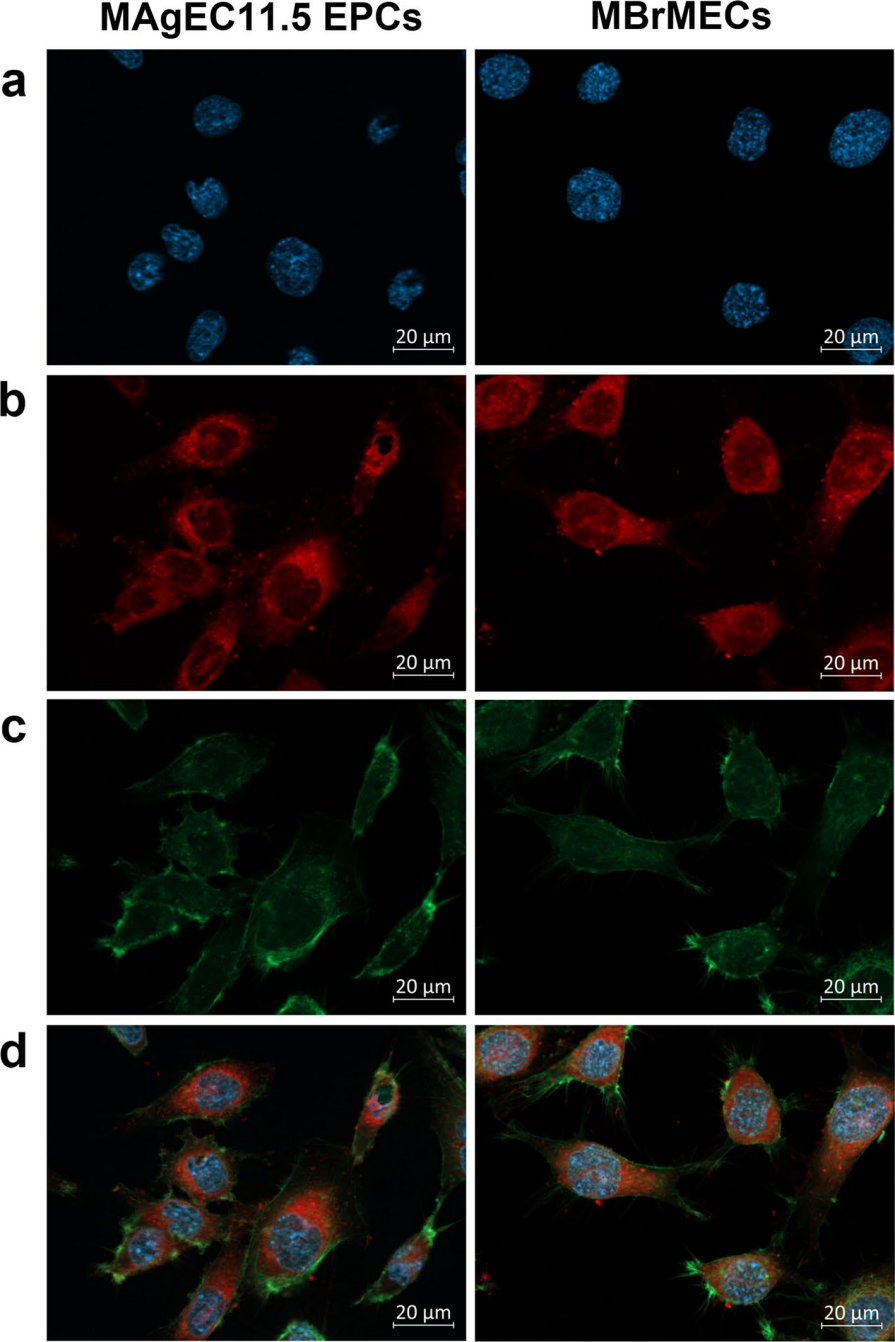
Exemplary fluorescence images of Endothelial Progenitor MAgEC11.5 and Brain Endothelial MBrMEC cells stained with **a** Hoechst 33342 (nuclei), **b** Dil (lipophilic membranes), and **c** Phalloidin-Atto 488 (F-actin) dyes. **d** Merged images. Magnification 60×

Fluorescence microscopy was first employed to visualize morphological features of both endothelial progenitor and brain endothelial cells (Fig. 1). For this purpose, we stained nuclei, cytoplasmic membranes of organelles, and actin filaments with Hoechst, Dil, and Phalloidin dyes, respectively. A visual inspection of the fluorescence images indicates no significant differences in the shape and size of both cells and their organelles. All cells displayed an elongated shape (up to 30-40 μm) with a properly structured cytoskeleton and oval-shaped nuclei of similar size (ca. 10 μm). The cytoplasm is rich in lipophilic membranes of organelles and lipid bodies. No specific staining dyes, even combined with antibodies, provide discrimination of both cell lines.

### A protocol of spectroscopic investigations of single cells

**Fig. 2:**
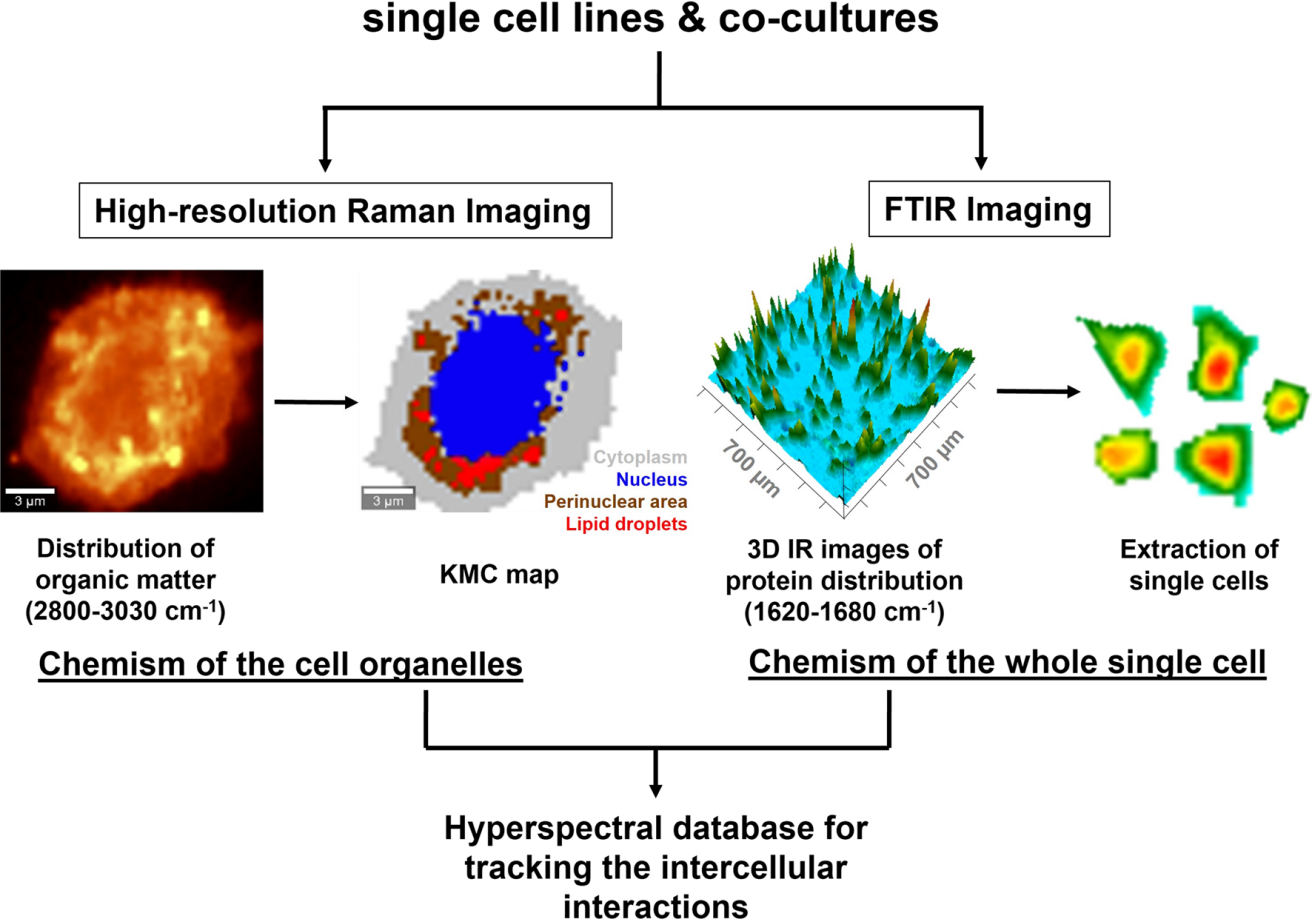
Schematic displaying the workflow of Raman and FTIR spectroscopy imaging of the studied cells

To investigate differences between the endothelial progenitor cells and brain endothelium together with their co-cultures, Raman and FTIR spectroscopy imaging was employed. With the achievable high spatial resolution of 0.3 µm for Raman microscopy with the use of the 532 nm laser excitation, it was possible to examine cells at a subcellular level. The biochemical composition of the cellular structures is coded in the spectrum and was firstly extracted as false-color distribution images constructed from integral intensities of marker bands of biomolecules (Fig. 2). Through k-mean cluster analysis (KMCA) of hyperspectral images, spectra revealing a similar chemical profile were grouped. In that way, the Raman image is segregated into classes with Raman characteristics of cell compartments, c.f. Fig. 2. The similar pixel spectra in the classes are next averaged giving us the data sets of mean Raman spectra. Afterward, the same samples were imaged utilizing FTIR-FPA microscopy taking several single snapshots from the area of 700 µm × 700 µm and screening tens cells at once. Rapid data collection from a large area but with a worse spatial resolution than in the Raman image (max. ca. 5.5 µm) delivers the chemical information of the whole single cell. To do it, we selected IR spectra of the cells with the highest signal-to-noise ratio (S/N), and pixel spectra were averaged (Fig. 2). Using both microscopic techniques, hyperspectral databases containing information about the chemism of single cell lines and their co-cultures at the cellular and subcellular level were established.

### Unique spectral features of endothelial progenitor MAgEC11.5 and brain endothelial MBrMEC cells

**Fig. 3:**
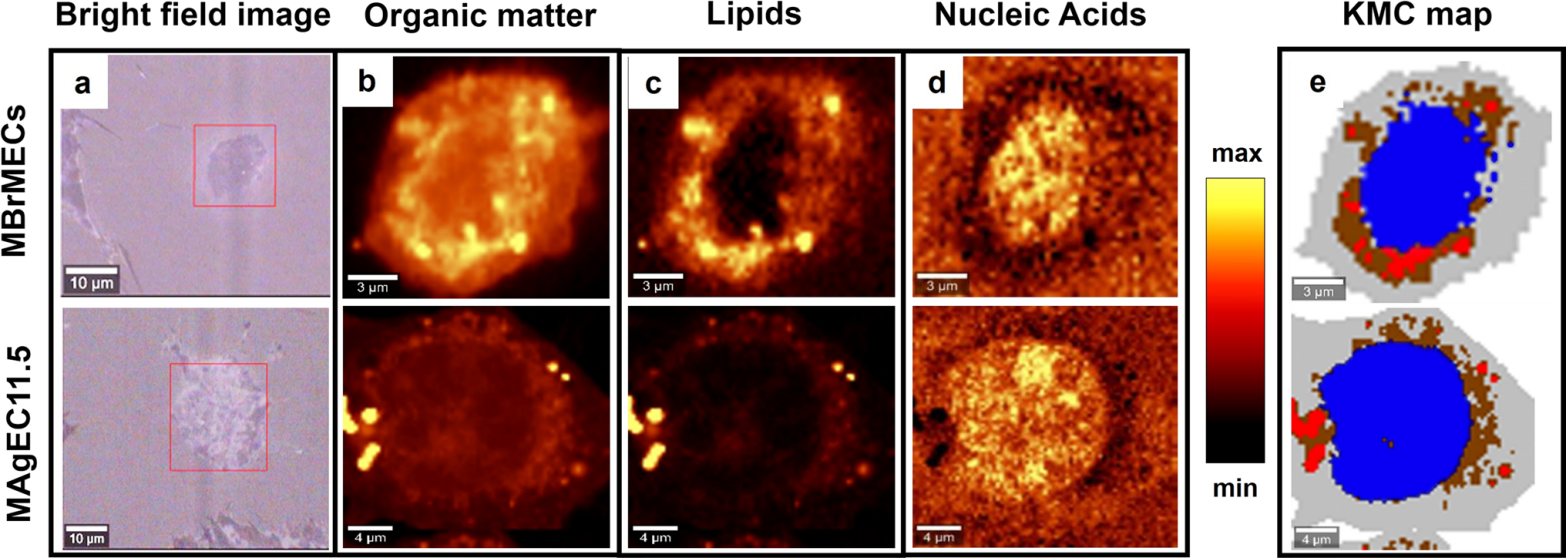
Bright-field (**a**) and distribution Raman images (**b**-**d**) of single brain endothelial (MBrMEC) and endothelial progenitor (MAgEC11.5) cells collected with a step size of 0.3 µm. The distribution of main macromolecules was calculated for organic matter (integration region: 2800-3030 cm^−1^), lipids (integration region: 2830-2900 cm^−1^), and nucleic acids (integration region: 790-810 cm^−1^). The corresponding false-color KMC maps (**e**) reveal the presence of the main subcellular compartments: cytoplasm (gray), nucleus (blue), perinuclear area (brown), and lipid droplets (red)

High-resolution Raman images of individually cultured Endothelial Progenitor MAgEC11.5 and Brain Endothelial MBrMEC cells collected with a 0.3 µm step size showed the presence of the main cell compartments (nucleus and cytoplasm) accompanied by accumulation of lipids in the cytoplasm (Fig. 3b-d). Based on KMCA, we found four classes of different Raman profiles which were assigned to the cytoplasm, nucleus, perinuclear area, and lipid droplets (Fig. 3e). To fasten Raman imaging measurements from 60 to 6 min per single cell, the step size of Raman imaging was increased from 0.3 up to 1 µm and KMCA still segregated the cell into these classes (Table S1 in SI). The averaged Raman spectra extracted from KMC classes of the endothelial progenitor and brain endothelial cells are displayed in Fig. S1 in SI. Band assignments to biomolecules are summarized in Table S2 in SI.

The average Raman spectra of subcellular compartments differ significantly. As cytoplasm consists of water, organic compounds like proteins, lipids, carbohydrates, and free amino acids, its spectral profile includes characteristic bands of these biomolecules (Fig. S1a and Table S2 in SI). The Raman spectra of the MBrMEC and MAgEC11.5 cytoplasm show typical features of proteins (1656, 2850-2965 cm^-1^) and lipids (1261, 1451, and 2850-3015 cm^-1^). Bands assigned to cholesterol esters (611 cm^-1^), amino acids (537, 647, 1009, and 1175 cm^-1^), nucleic acids (729, 790, 1099, 1318, and 1340 cm^-1^), cytochromes (755, 1585 cm^-1^), and phospholipids (1132 cm^-1^) are also present. The class of nucleus was segregated in the cell based on an increased intensity of Raman bands of nucleic acids (790, 1099, 1340, and 1381 cm^-1^), c.f. Fig. S1b and Table S2 in SI. Apart from the characteristic signal of the genetic material, signals of proteins (1621 cm^-1^), amino acids (537, 857 cm^-1^), and phospholipids (1132 cm^-1^) appear due to the composition of the cell nucleus and its membrane [35].

Cytochromes play an important role in the proper functioning of mitochondria and endoplasmic reticulum (ER) and their concentration in these cellular compartments is significantly higher. Their increased resonance Raman signals at 755 and 1585 cm^-1^ contributed to distinguishing the perinuclear area (Figs. 3e and S1c in SI) [36]. The lipid-rich nature of this region was another feature identified by the intensive signals at 1306, 1660, and 2853 cm^-1^ while phospholipids in the membranous ER structure were manifested by the 1132 cm^-1^ band. An increased protein-lipid content was demonstrated by a highly intense 2850-3015 cm^-1^ region similar to the cytoplasm. Raman imaging also revealed the accumulation of lipids in the form of droplets (LDs) observed by us previously in endothelial, cancer, and blood cells (Figs. 3e and S1d in SI) [16, 18, 36]. The Raman spectrum shows a typical signature of lipids with the intensive high-wavenumber region (2850-3015 cm^-1^) and numerous bands of fatty acids (FAs) acyl chain (1070, 1306, and 1451 cm^-1^), unsaturated moieties in fatty acids (UFAs) (1261, 1660, and 3013 cm^-1^), cholesterol esters (611, 706 cm^-1^), triacylglycerols (TAGs) (1740 cm^-1^), and the phospholipid membrane (1132 cm^-1^).

The calculated difference spectra between the cell lines unambiguously show that differentiated MBrMEC and progenitor MAgEC11.5 cells exhibit unique Raman signatures in each structure organization in the sense of an increased content of a given class of biomolecules (Fig. S1 in SI, Table 1). Briefly, the MBrMEC cytoplasm is rich in cytochromes, phosphate-containing molecules, and lipids, whereas the cytoplasm in MAgEC11.5 EPCs stores cholesterol, long-chain fatty acids, nucleic acids, and an increased level of proteins. The difference spectrum of the nuclei indicates a greater accumulation and/or condensation of the genetic material in the brain endothelium than in the progenitor cells. Chromatin in MBrMEC is additionally composed of proteins with β-sheet conformation. In turn, the nuclei of MAgEC11.5 EPCs contain additional fatty acids. The perinuclear area of both cell lines revealed two distinct biochemical profiles. The dominant component of this cellular compartment in brain endothelium are lipids with long-acyl chains and the C=C group. On the other hand, the nuclei in the progenitor cells are surrounded by the matrix containing a higher level of proteins rich in Tyr and Phe amino acid residues and cytochromes, nucleic acids, and cholesterol. LDs also differ by the content of TAGs, saturation degree of FAs, cholesterol, and phospholipids (Table 1). In addition, LDs in progenitor cells are characterized by Raman bands assigned to phenylalanine. Interestingly, the number of lipid droplets also varied between the cell types. They occurred in most of the MAgEC11.5 cells (81%) whereas only 63% of MBrMECs showed their presence (Table S1 in SI).

**Table 1:** The Raman and FTIR bands specific for MBrMECs and MAgEC11.5 cells and determined from their difference spectra

	MBrMECs		MAgEC11.5 EPCs	
	Band position (cm^-1^)	Biomolecules	Band position (cm^-1^)	Biomolecules
Cytoplasm	647 748, 1132, 1318,1585 1095, 1244 1451	Tyr Cytochromes PO_2_-molecules Lipids	537, 1654, 2927 611 2850 729, 790 1340	Proteins Cholesterol Long FAs Nucleic acids Organics
Nucleus	1381, 1585 790, 1099, 1344, 2960 1251, 1670	A, G Nucleic acids Antiparallel β-sheets	729 537, 1448, 2882, 2927 2853	A Proteins Long FAs
Perinuclear area	1451, 2879 2853 3013	Lipids Long FAs UFAs	537, 647, 1009, 1175 1650, 2930 755, 1132, 1585 611 729, 790, 1101, 1244	Tyr, Phe Proteins Cytochromes Cholesterol Nucleic acids
Lipid droplets	1070, 1451, 2895 1306, 2853 1660, 3013 1749	Lipids Long FAs UFAs Unsaturated TAGs	537, 1009 611, 706 1132, 2962	Phe Cholesterol Phospholipids
Whole cell	1681, 1645, 1619, 1545, 1458 1085, 1050	Proteins: β-turns, α-helices, β-sheets DNA	1666 2851, 2933, 1388 1067 1146, 1108	Proteins: 3_10_-helices Long chain FAs Cholesterol esters Lactate, poly/sugars

*A* adenine, *C* cytosine, *G* guanine, *T* thymine, *U* uracil, *Tyr* tyrosine, *Phe* phenylalanine, *FAs* fatty acids, *UFAs* unsaturated fatty acids, *TAGs* triacylglycerols

FTIR spectra delivered the biochemical information from the whole single cells, mainly from the nucleus and cytoplasm (Figs. 2, S2a, b, Table S3 in SI). A visual inspection of the second derivative FTIR spectra of both cell lines indicates the presence of proteins (1458−1681 cm^-1^), nucleic acids (1085, 1122, and 1238 cm^-1^), and carbohydrates (1050 cm^-1^). Vibrations of lipids are also observed, and they indicated classes, such as cholesterol esters (1166, 1725 cm^-1^), phospholipids (1332 cm^-1^), fatty acids (1396, 1710, and 2855 cm^-1^), and triacylglycerols (1746 cm^-1^). All the biocomponents contribute to spectral differences of the cell lines (Fig. S2c, Table 1). The dominant difference between the cell lines is a set of proteins of various secondary structures. The spectral profile of progenitor cells is dominated by the 1666 cm^-1^ band assigned to 3_10_-helix conformation whereas the overall protein composition of MBrMECs is manifested by α-helices (1645 cm^-1^), β-turns (1681 cm^-1^), and intermolecular protein aggregates (1619 cm^-1^). The enhanced cellular metabolism involving sugars and lactate is observed in the case of the progenitor cells (1146, 1108 cm^-1^). The IR markers of lipids and nucleic acids confirm the observations from the Raman spectra (Table 1).

**Fig. 4:**
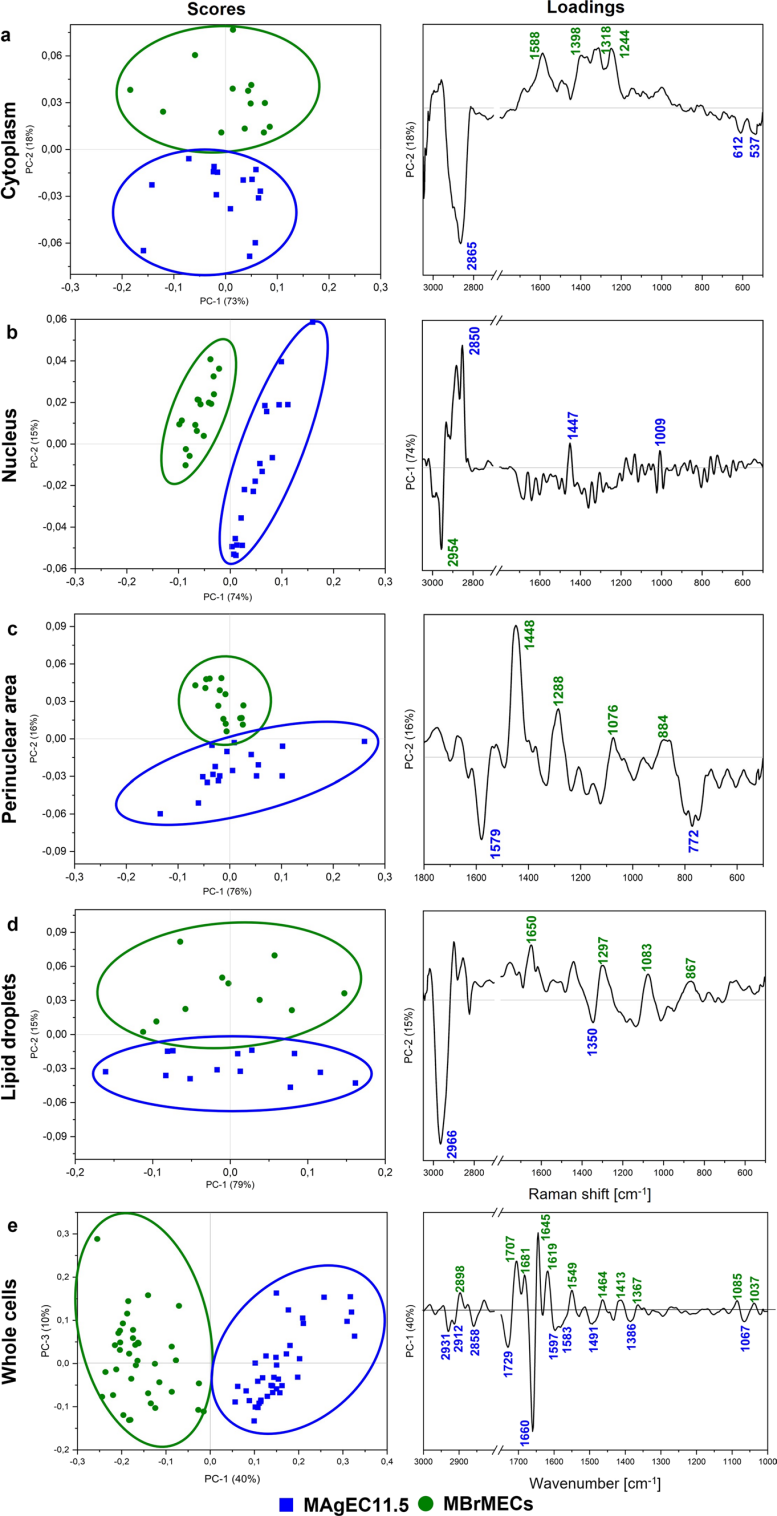
The scores (left) and loadings (right) plots from principal component analysis performed on Raman spectra of their cellular compartments (3050-500 cm^-1^) and FTIR spectra of the whole MAgEC11.5 EPCs and MBrMEC cells (3000-1000 cm^-1^): **a** cytoplasm, **b** nucleus, **c** perinuclear area, **d** lipid droplets, **e** whole cells. The fingerprint region (below 1800 cm^-1^) is shown only for the perinuclear area, because of the lack of high loading vectors in the high-wavenumber region (data not shown). Each point refers to a single cell

Next principal component analysis (PCA) was used to discriminate the cell lines cultured separately in an unsupervised way. PCA delivers two types of information. Score plots show the separation of the experimental groups while loading plots display the spectral discriminators (spectral biomarkers) which contribute to this segregation but are often hidden in a typical examination of the spectra. Here, the PCA of the spectral data shows clear-cut discrimination between MBrMECs and MAgEC11.5 cells regardless of the imaging technique and the level of cellular organization (Fig. 4). The scree plots for each PCA segregation can be found in SI (Fig. S3).

The total biochemical composition of both cells lines coded in the IR spectra is differentiated along PC-1 with a high variance of 40% (Fig. 4e). The main discriminators are observed at 1619, 1645, 1660, 1681, 1707, and 1729 cm^-1^, and they originate from cellular compartments containing proteins, fatty acids, and triacylglycerols, respectively (Table S2 in SI). In turn, the PCA scores plot calculated for the Raman spectra indicates that the highest variance of the MBrMECs vs progenitor ECs discrimination (PC-1, 74%) was achieved for the nuclei (Fig. 4b). The loadings graph highlights the contribution of the vectors assigned to lipids (2850 and 2954 cm^−1^) and proteins (1447 cm^-1^) with high content of phenylalanine (1009 cm^-1^). Other cellular compartments (cytoplasm, perinuclear area, and lipids droplets) of both cell lines also possess unique properties which can contribute to their identification. For each of them, the progenitor and the differentiated ECs were well differentiated along PC-2 with a variance of ca. 15-18% (Fig. 4a, c, d). In the case of cytoplasm, the lipidic (particularly, cholesterol) and the proteinaceous (Phe) signals at 2865, 612, and 537 cm^-1^ discriminated MAgEC11.5 from MBrMECs in that the contributing variables are assigned to nucleic acids (1588, 1398, 1318, and 1244 cm^-1^) (Fig. 4a). The loading peaks of the perinuclear area are different than in the cytoplasm and are assigned to pyrimidine bases in MAgEC11.5 cells (1579 and 772 cm^-1^) and lipids (1448, 1288, and 1076 cm^-1^) with proteins (884 cm^-1^) in MBrMECs (Fig. 4c). The segregation of the lipid droplets results from differences in their composition, i.e., LDs in MAgEC11.5 cells specifically contain lipids with branched acyl chain (2966 and 1350 cm^-1^), whereas the brain endothelium produces LDs with FAs and TAGs with the unsaturated acyl chains (1297, 1083, and 867 cm^-1^) mixed with proteins (1650 cm^-1^) (Fig. 4d). The PCA results confirm that the spectral differences between the brain endothelium and progenitor cells are statistically significant, and the defined-above marker bands are unique contributors to the separation of the cell lines at the level of the whole cells and their cellular compartments when they culture individually.

### Tracking intercellular interactions between whole MAgEC11.5 EPCs and MBrMEC cells

**Fig. 5:**
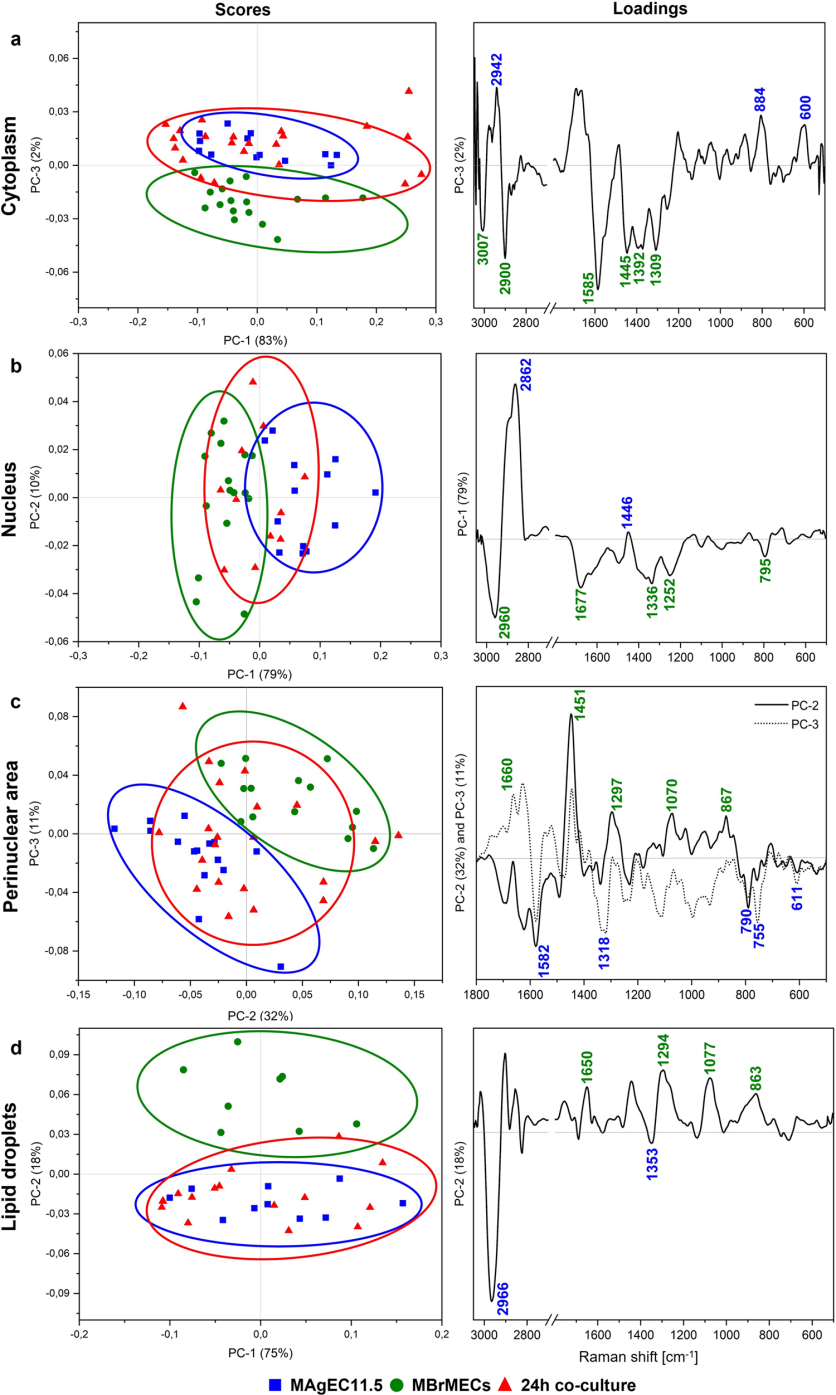
The scores plots (left) and loadings (right) from PCA analysis performed on Raman spectra of cellular compartments of MAgEC11.5 EPCs and MBrMEC cells, and their 24-h co-cultured cells. Each point refers to a single cell

**Fig. 6:**
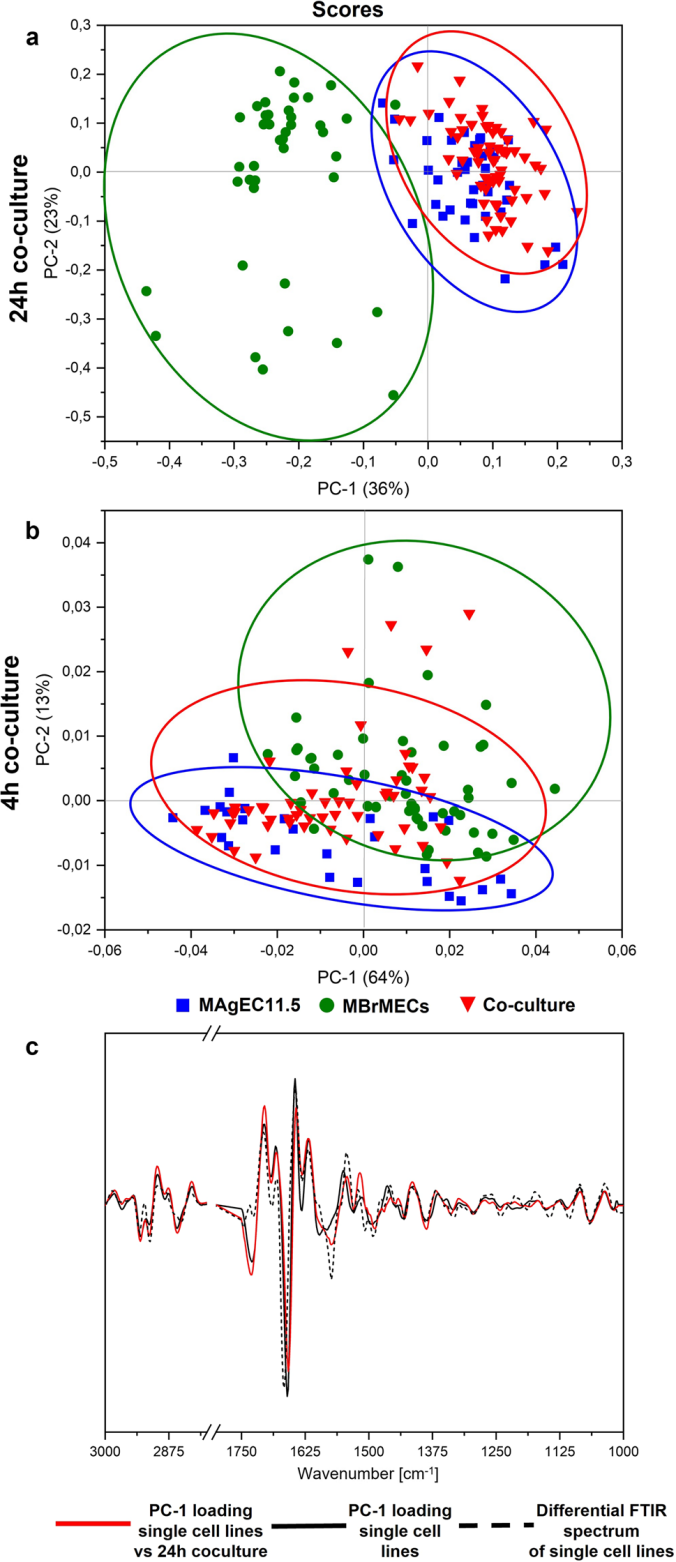
The PCA scores plots for **a** 24-h and **b** 4-h co-cultures of whole MAgEC11.5 EPCs and MBrMEC cells together with **c** PC-1 loading graphs of the 24-h co-culture and individual cells lines (from Fig. 4e) compared with the difference spectrum (from Fig. S2). Data analysis was performed for the whole cells acquired in FTIR spectroscopy imaging

To further observe the direct contact between the whole MAgEC11.5 EPCs and MBrMEC cells and determine its effect on genomic, proteomic, or lipidomic features, both cell types were co-cultured in a ratio of 1:1 for 24 h. Cells were randomly selected and imaged by Raman and FTIR spectroscopy in the same way as for the individual cell lines and then PCA analysis was performed to assess firstly similarities/differences between MBrMECs, MAgEC11.5 cells, and the effect of their co-culture. The loading graphs also indicate whether the new dataset added to the chemometric analysis affects the spectral discriminators contributing to the discrimination. This means the alternation of the metabolic routes in the co-cultured cells. The results of the PCA analysis are displayed in Figs. 5 and 6 with scree plots depicted in SI (Fig. S4).

High-resolution Raman imaging combined with KMCA also exhibited the presence of the cytoplasm, nucleus, perinuclear area, and lipids bodies in the co-cultured cells as it was shown for the cell lines (Fig. 3). Among these compartments, the cytosol is the primary site for most enzymatic reactions and metabolic activity of the cellular machinery and should reveal firstly metabolic exchange between co-cultured cells. The Raman-based scores plot of PCA shows the grouping of the cytoplasm of the co-cultured cells with the progenitor cells and their separation from the brain endothelium along the PC-3 axis (Fig. 5a). Two of the spectral discriminators in the loadings plot appeared at similar positions as for the PCA analysis of the individual whole MAgEC11.5 EPCs and MBrMEC cells groups (1588 and 1392 cm^-1^ for brain endothelium) and they represent nucleic acids with a high contribution of A and G. New PCA vectors of proteins and TAGs are observed in the group of the EPC/co-cultures cells (2924, 884, and 600 cm^-1^), while the brain endothelial cells’ variables reveal the contribution of unsaturated lipids, FAs, and TAGs (3007, 2900, 1445, and 1309 cm^-1^) to the grouping of these cells. In the case of the nuclei, we observe first the differentiation of separately cultured whole MAgEC11.5 EPCs and MBrMEC cells at the same level of variance (79% along the PC-1) as previously, whereas the nuclei of the cells interacting with each other for 24 h were spread evenly among the MAgEC11.5 EPC (*ve*+) and MBrMEC (*ve*–) groups, cf. Figs. 4b and 5b. A similar distribution of the experimental groups is established for the perinuclear area (Fig. 5c). We also note that cell lines are grouped along PC-2 and PC-3 axes with higher variance (32% and 11%, respectively) than in the PCA calculated for the single cell lines only (Fig. 4c). The loading plots show new vectors attributing to cytochromes (1582 and 755 cm^-1^), guanine (1318 cm^-1^), and cholesterol (611 cm^-1^) in the progenitor group, while the perinuclear area of brain endothelial cells is additionally characterized by the signals of unsaturated and saturated FAs (1660, 1297, and 867 cm^-1^). Interestingly, the co-cultured cells are distinctly assigned to the EPC group based on the Raman features of lipid droplets along PC-2 (18% of variation) and the loading graph is identical to the one determined for the separately cultured cell lines, see Figs. 4d and 5d. To confirm this PCA result, we calculated the intensity ratio of the Raman bands assigned to the C=C and CH_2_ groups which is a well-established tool to estimate the unsaturation degree of the acyl chain in lipids [19, 20]. The number of C=C bonds in the LD lipids of EPCs and the co-culture is identical and lower than in LDs of the brain endothelium (Fig. S5 in SI).

FTIR spectra of the whole cells also indicated the similarity of cellular chemism between the 24-h co-cultured cells and the endothelial progenitor cells as presented by the PCA scores plot (PC-1, 36% of variation), c.f. Fig. 6a. Due to fast measurements using FTIR imaging, we examined also negative controls. Here, MBrMEC and MAgEC11.5 cells in the 1:1 ratio were co-cultured for 4 h only. The PCA scores plot shows that these cells are not assigned to the progenitor cells group and are evenly distributed between both cell lines (Fig. 6b). Finally, we compared the PC-1 loading graphs of the co-cultured and separately cultured cell lines with the difference trace of FTIR spectra of MBrMECs and MAgEC11.5 EPCs (Fig. 6c). All three IR-based traces are almost identical what additionally confirms our observation that the brain endothelium adapts features of the progenitor cells due to their direct interactions within 24 h. The PCA analysis of the Raman spectra indicates in turn that these interactions significantly alter the cytoplasmic metabolism including the synthesis of lipid droplets.

**Fig. 7:**
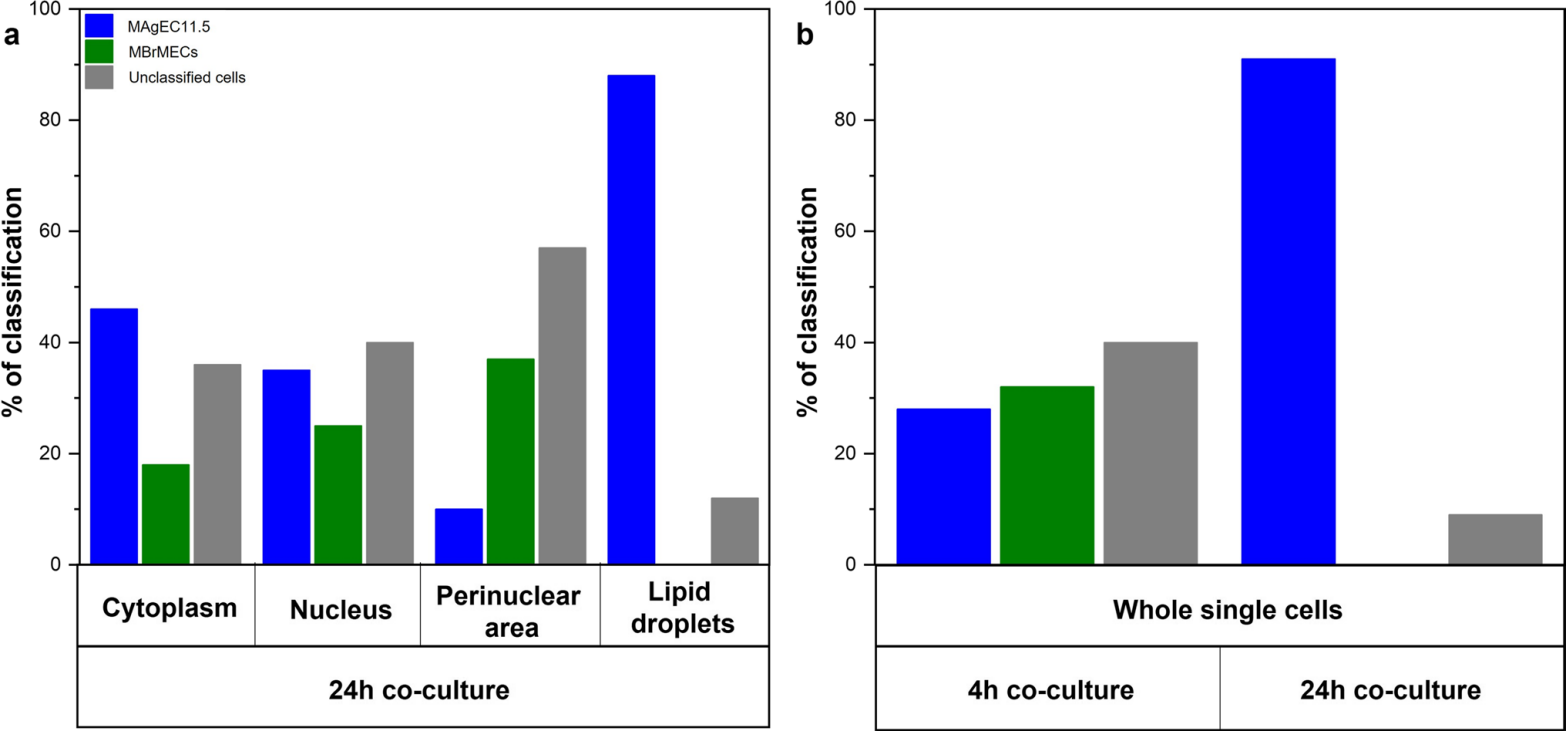
Bar chart with the % classification of the 24-h co-cultured cells to whole MAgEC11.5 EPCs and MBrMEC cells based on the **a** cellular compartment – cytoplasm, cell nucleus, endoplasmic reticulum, and lipid droplets (high-resolution Raman imaging) and **b** the whole cells (FTIR imaging) compared with negative control

In addition, we performed a supervised chemometric method – partial least square regression (PLRS) to verify the classification power of Raman and FTIR spectra for the identification of the re-programmed cells at various levels of the cellular structure. Regression models for the calibration and prediction sets had correlation coefficients above 0.9, which showed that the datasets were well modeled (Table S4 and Figs. S6-8 in SI). Figure 7 summarizes the prediction rates which include the assignment of the co-cultured cells to three groups whole MAgEC11.5 EPCs, MBrMEC, and cells unclassified to either of the cell lines. We assume that the latter may represent the cells of different phenotypes than the model (further, we call them UCCs - unclassified). In the case of the Raman spectra of cytoplasm and nucleus, a similar number of the cells (ca. 40%) are classified as endothelial progenitor cells and UCCs. Only 20-30% of cells preserved the features of the brain endothelium. Over 50% of co-cultured cells (UCCs) reprogrammed the biochemical composition of the perinuclear area into a new functionality whereas 40% of cells showed a spectral profile like MBrMECs. Only for the class of lipid droplets, the co-cultured cells are assigned to the endothelial progenitor cells (ca. 90%). None of the cells from the prediction group was classified as the brain endothelium. The same result was obtained for the FTIR spectra of the whole cells. The prediction model used for the negative control classified the cells into all prediction groups.

## Discussion

The used here Raman and FTIR imaging deliver complementary results giving an insight into the chemism of the individual cell lines at the cellular and subcellular level (Fig. 2). We established the label-free protocol how to investigate the cells to achieve this chemical complementarity, including the measurement and analysis steps. We showed that this approach recognizes the genetic and metabolic differences between the MAgEC11.5 EPCs and MBrMEC cells even though there are no known specific biomarkers yet and standard bioimaging techniques show only morphological features (Fig. 1).

From the biological point of view, high-resolution Raman imaging would be certainly a more acceptable tool because of its similar spatial resolution to fluorescence microscopy (Figs. 1, 3). We showed that one can determine the spectral biomarkers of the endothelial and progenitor cells in nuclei, cytoplasm, perinuclear area, and lipid droplets without using labels and they enabled their further unsupervised discrimination (Fig. 4, Table 1). The PCA analysis and difference spectra indicated a few discriminators that contributed to the significant discrimination of the nuclei in the separately cultured brain endothelium and endothelial cells (PC-1, 74%; Fig. 4b and Table 1). MAgEC11.5 cells exhibited a higher contribution of long FAs and proteins contrary to MBrMECs in that a typical nucleus material was identified. It is well known that the nuclear envelope (NE) surface constantly increases during interphase in cells of high proliferative potential like EPCs [37]. This requires a continuous synthesis of nuclear pore complexes and other NE components. Most cellular lipid synthesis occurs at the ER and NE interface and thus lipids can freely diffuse through this membrane continuum. For this reason, probably, the spectral profile of the nucleus in the progenitor cells was dominated by lipids. Furthermore, TEM images of the EPC cytoplasm show a high abundance of cellular organelles like mitochondria, rough endoplasmic reticulum, and Golgi complex that are responsible for the significant protein synthesis capability of the progenitor cells [38]. Thus, they can secrete many growth factors to maintain their growth and differentiation [39–41]. The Raman spectra indicated that the perinuclear area of the progenitor cells is, in fact, richer in cytochromes and accompanied by other proteins and nucleic acids whereas this cellular compartment in MBrMECs contains long fatty acids and unsaturated lipids synthesized in the smooth endoplasmic reticulum (Table 1). This chemical composition can indicate a higher contribution of rough and smooth ER of MAgEC11.5 EPCs and MBrMEC cells, respectively, since staining with the Dil dye did not indicate substantial differences between the cell lines (Fig. 1). On the other hand, ca. 80% and 60% of the investigated MAgEC11.5 EPCs and MBrMEC cells, respectively, produced lipid droplets but of a different composition (Fig. 3, Table S1). More unsaturated fatty acids and their TAGs were synthesized by the brain endothelium, whereas LDs in the progenitor cells additionally contained cholesterol and phospholipids. In both cases, the unsaturation degree was not high and did not exceed the value for linoleic acid (18:2) (Fig. S3). To our best knowledge, there is no study published so far that shows the presence of lipid droplets in the brain endothelium and progenitor cells.

The biochemical composition of all intercellular compartments was expressed by the FTIR spectrum of the single cell (Fig. 2). Since IR imaging is much faster than Raman microscopy, one can employ this technique for the rapid examination of chemical alternations/differences in cells and the classification purpose like in this work. The PCA-based separation of cell lines confirmed the Raman spectroscopy results with a high variation of 40% (Fig. 4e) while the determined spectral markers indicated pronounced differences in protein conformations (Table 1). The recognition of secondary structures of these fundamental biomolecules is the well-known advantage of FTIR spectroscopy. We revealed here that proteins containing 3_10_-helices were specific for the progenitor cells, while intermolecular aggregates of β-sheets, α-helices and, β-turns were more abundant in brain endothelium. Since 3_10_-helices in cellular proteins were proposed to be intermediates in the folding/unfolding of α-helices, we suggest that their higher contribution in the progenitor cells might be associated with an enhanced cytoskeleton reorganization due to their potential for proliferation [42, 43]. A lipid nature of the EPC cells was also confirmed, and additionally, bands of poly/sugars and lactate may exhibit the amplified glycolytic process (Table 1).

Both vibrational spectroscopy microscopies also showed for the first time cell-to-cell interactions between the brain endothelium and the progenitor endothelial cells (Figs. 5, 6). The FTIR-based analysis of the whole intercellular interior indicated reprograming of MBrMEC into MAgEC11.5 EPCs with a full transfer of the biochemical properties of the progenitor cells in 90% of the co-cultured cells (Figs. 6, 7). While the analysis of Raman signals from their cellular compartments and their PLSR classification suggested that the cell-cell interactions transform the metabolism of the cytoplasm and the organelles located around the nucleus (Figs. 5, 7). Only LDs were synthesized with the chemical composition of the cell progenitors (Fig. S3), whereas the nuclei preserved the properties of the cell lines. The new markers of the cytoplasm in the co-cultured cells were observed in the lipid classes (TAGs, un-/saturated FAs) which implicates the amplification and/or transformation of their synthesis routes. Although the PCA analysis of the perinuclear area did not exhibit the assignment of the co-cultured cells to one of the cell lines, the appearance of the discriminators associated with cytochromes and fatty acids implicated that the alternation of their biochemical processes must have occurred and it is confirmed by the high number of the interacting cells unclassified in PLSR (Figs. 5, 7).

## Conclusion

The progenitor endothelial cells have been broadly investigated to assess their clinical significance in diseases associated with brain endothelial dysfunction due to their ability to differentiate toward mature endothelial cells. However, the mechanisms of their direct interactions and effects on the treatment of neurodegenerative diseases are still poorly understood and the wide and comprehensive evaluation is highly in demand. Our work showed the proof of principles that label-free imaging tools sensitive to molecular vibrations recognize the cells of similar phenotypes and their direct interactions. While the RS technique provided the chemical description of metabolic differences of single cells, FTIR imaging gave an insight into the structural diversities of the same samples. We propose that both modalities offer an attractive strategy for tracking cellular interactions without a need for the specific labeling that is particularly important when biomarkers are not known or they do not differ between investigated specimens. The obtained spectral datasets can be easily combined with unsupervised multivariate analysis and prediction models to identify the cell communication and junction which induce further new physiological processes. The interpretation of the spectral markers suggested that the brain endothelium affected by the progenitor cells primarily triggers metabolic pathways in the cytoplasm which are responsible for the synthesis of lipid droplets. Further extensive work is required to confirm these observations and validate the applicability of vibrational spectroscopy to track cell-cell interactions. Furthermore, a prolonged intercellular interactions would be interesting to systematically assess the evolution of the final resulting cells in the real microenvironment.

### Supplementary Information

Below is the link to the electronic supplementary material.

Supplementary file1 (DOCX 2446 KB)

## Data Availability

The datasets generated during and/or analyzed during the current study are available from the corresponding author on reasonable request.

## References

[1] Hristov M, Erl W, Weber PC (2003). Endothelial progenitor cells: isolation and characterization. Trends Cardiovasc Med.

[2] Chopra H, Hung MK, Kwong DL (2018). Insights into endothelial progenitor cells: origin, classification, potentials, and prospects. Stem Cells Int.

[3] Li J, Ma Y, Miao XH (2021). Neovascularization and tissue regeneration by endothelial progenitor cells in ischemic stroke. Neurol Sci.

[4] Rudnicka-Drożak E, Drożak P, Mizerski G, Drożak M (2022). Endothelial progenitor cells in neurovascular disorders—a comprehensive overview of the current state of knowledge. Biomedicines.

[5] Fan Y, Shen F, Frenzel T (2011). Endothelial progenitor cell transplantation improves long-term outcome in mice. Ann Neurol.

[6] Fang J, Guo Y, Tan S (2019). Autologous endothelial progenitor cells transplantation for acute ischemic stroke: a 4-year follow-up study. Stem Cells Transl Med.

[7] Zhen-Zhou C (2023) Autologous endothelial progenitor cells transplantation for chronic ischemic stroke. https://clinicaltrials.gov/ct2/show/NCT02605707. Accessed 12 June 2023

[8] Thinard R, Farkas AE, Halasa M (2022). “Endothelial antibody factory” at the blood brain barrier: novel approach to therapy of neurodegenerative diseases. Pharmaceutics.

[9] Ben-Shoshan J, Keren G, George J (2006). Endothelial progenitor cells (EPCs)—new tools for diagnosis and therapy. Harefuah.

[10] Tang Y-H, Ma Y-Y, Zhang Z-J (2015). Opportunities and challenges: stem cell-based therapy for the treatment of ischemic stroke. CNS Neurosci Ther.

[11] Ramm Sander P, Hau P, Koch S (2013). Stem cell metabolic and spectroscopic profiling. Trends Biotechnol.

[12] Collet G, Szade K, Nowak W (2016). Endothelial precursor cell-based therapy to target the pathologic angiogenesis and compensate tumor hypoxia. Cancer Lett.

[13] Klimkiewicz K, Weglarczyk K, Collet G (2017). A 3D model of tumour angiogenic microenvironment to monitor hypoxia effects on cell interactions and cancer stem cell selection. Cancer Lett.

[14] Bizourne N, Denis V, Legrand A (1993). A SV-40 immortalized murine endothelial cell line from peripheral lymph node high endothelium expresses a new α-l-fucose binding protein. Biol Cell.

[15] Kieda C, Paprocka M, Krawczenko A (2002). New human microvascular endothelial cell lines with specific adhesion molecules phenotypes. Endothel J Endothel Cell Res.

[16] Kujdowicz M, Placha W, Mech B (2021). In vitro spectroscopy-based profiling of urothelial carcinoma: a fourier transform infrared and raman imaging study. Cancers (Basel).

[17] Wiercigroch E, Staniszewska-Slezak E, Szkaradek K (2018). FT-IR spectroscopic imaging of endothelial cells response to tumor necrosis factor-α: to follow markers of inflammation using standard and high-magnification resolution. Anal Chem.

[18] Szafraniec E, Wiercigroch E, Czamara K (2018). Diversity among endothelial cell lines revealed by Raman and Fourier-transform infrared spectroscopic imaging. Analyst.

[19] Pacia MZ, Majzner K, Czamara K (2020). Estimation of the content of lipids composing endothelial lipid droplets based on Raman imaging. Biochim Biophys Acta - Mol Cell Biol Lipids.

[20] Czamara K, Majzner K, Selmi A (2017). Unsaturated lipid bodies as a hallmark of inflammation studied by Raman 2D and 3D microscopy. Sci Rep.

[21] Usoltsev D, Sitnikova V, Kajava A, Uspenskaya M (2019). Systematic FTIR spectroscopy study of the secondary structure changes in human serum albumin under various denaturation conditions. Biomolecules.

[22] Grewal MK, Huppertz T, Vasiljevic T (2018). FTIR fingerprinting of structural changes of milk proteins induced by heat treatment, deamidation and dephosphorylation. Food Hydrocoll.

[23] Wang J, Ma X, Yu Z (2018). Studies on thermal decomposition behaviors of demineralized low-lipid microalgae by TG-FTIR. Thermochim Acta.

[24] Traoré M, Kaal J, Martínez Cortizas A (2016). Application of FTIR spectroscopy to the characterization of archeological wood. Spectrochim Acta Part A Mol Biomol Spectrosc.

[25] Xiong Q, Hu LX, Liu YS (2019). New insight into the toxic effects of chloramphenicol and roxithromycin to algae using FTIR spectroscopy. Aquat Toxicol.

[26] Tott S, Grosicki M, Glowacz J (2021). Raman imaging-based phenotyping of murine primary endothelial cells to identify disease-associated biochemical alterations. Biochim Biophys Acta Mol Basis Dis.

[27] Indari O, Tiwari D, Tanwar M (2022). Early biomolecular changes in brain microvascular endothelial cells under Epstein-Barr virus influence: a Raman microspectroscopic investigation. Integr Biol.

[28] Choi JS, Ilin Y, Kraft ML, Harley BAC (2018). Tracing hematopoietic progenitor cell neutrophilic differentiation via Raman spectroscopy. Bioconjug Chem.

[29] Hsu CC, Xu J, Brinkhof B (2020). A single-cell Raman-based platform to identify developmental stages of human pluripotent stem cell-derived neurons. Proc Natl Acad Sci USA.

[30] Germond A, Panina Y, Shiga M (2020). Following embryonic stem cells, their differentiated progeny, and cell-state changes during iPS reprogramming by Raman spectroscopy. Anal Chem.

[31] Mata-Miranda MM, Vazquez-Zapien GJ, Rojas-Lopez M (2017). Morphological, molecular and FTIR spectroscopic analysis during the differentiation of kidney cells from pluripotent stem cells. Biol Res.

[32] Heraud P, Ng ES, Caine S (2010). Fourier transform infrared microspectroscopy identifies early lineage commitment in differentiating human embryonic stem cells. Stem Cell Res.

[33] Lasch P. Cytospec^TM^. http://www.cytospec.com. Accessed on Jul 2022

[34] Bassan P, Kohler A, Martens H (2010). Resonant Mie scattering (RMieS) correction of infrared spectra from highly scattering biological samples. Analyst.

[35] Mimura Y, Imamoto N (2016) Nuclear organization (nuclear structure and dynamics), encyclopedia of cell biology, vol 2. Elsevier Ltd, pp 311–318

[36] Borek-Dorosz A, Grosicki M, Dybas J (2022). Identification of inflammatory markers in eosinophilic cells of the immune system: fluorescence, Raman and CARS imaging can recognize markers but differently. Cell Mol Life Sci.

[37] De Magistris P, Antonin W (2018). The dynamic nature of the nuclear envelope. Curr Biol.

[38] Kim Y, Kim TK, Shin Y (2021). Characterizing organelles in live stem cells using label-free optical diffraction tomography. Mol Cells.

[39] Wang Q, Zhang W, He G (2016). Method for in vitro differentiation of bone marrow mesenchymal stem cells into endothelial progenitor cells and vascular endothelial cells. Mol Med Rep.

[40] Amerion M, Valojerdi MR, Abroun S, Totonchi M (2018). Long term culture and differentiation of endothelial progenitor like cells from rat adipose derived stem cells. Cytotechnology.

[41] Lupu M, Khalil M, Iordache F (2011). Direct contact of umbilical cord blood endothelial progenitors with living cardiac tissue is a requirement for vascular tube-like structures formation. J Cell Mol Med.

[42] Miick S, Martinez G, Fiori W (1995). Short alanine-based peptides may form 3_10_-helices and not α-helices in aqueous solution. Nature.

[43] Armen R, Alonso DOV, Daggett V (2003). The role of α-, 3_10_- and π-helix in helix → coil transitions. Protein Sci.

