# Correction: Molecular tracking of interactions between progenitor and endothelial cells via Raman and FTIR spectroscopy imaging: a proof of concept of a new analytical strategy for in vitro research

**DOI:** 10.1007/s00018-023-05038-6

**Published:** 2024-01-28

**Authors:** Karolina Augustyniak, Aleksandra Pragnaca, Monika Lesniak, Marta Halasa, Agata Borkowska, Ewa Pieta, Wojciech M. Kwiatek, Claudine Kieda, Robert Zdanowski, Kamilla Malek

**Affiliations:** 1 Department of Chemical Physics, Faculty of Chemistry, Jagiellonian University in Krakow, Gronostajowa 2, Krakow, 30-387 Poland; 2 Doctoral School of Exact and Natural Sciences, Jagiellonian University in Krakow, Prof. S. Lojasiewicza 11, Krakow, 30-348 Poland; 3 Laboratory of Molecular Oncology and Innovative Therapies, Military Institute of Medicine-National Research Institute, Szaserow 128, Warsaw, 04-141 Poland; 4 Transplant Immunology, The Houston Methodist Research Institute, Houston, TX, USA; 5 Postgraduate School of Molecular Medicine, Medical University of Warsaw, Zwirki i Wigury 61, Warsaw, 02-091 Poland; 6 Institute of Nuclear Physics, Polish Academy of Sciences, Radzikowskiego 152, Krakow, 31-342 Poland; 7 Center for Molecular Biophysics, UPR4301 CNRS, Orleans, France; 8 Department of Surgery, The Houston Methodist Hospital, Houston, TX, USA

**Cellular and Molecular Life Sciences (2023) 80:329**

10.1007/s00018-023-04986-3

In this article all units were processed incorrectly and they have been corrected now.

The original article has been corrected.

