# Supplementary material for: Molecular tracking of interactions between progenitor and endothelial cells via Raman and FTIR spectroscopy imaging: a proof of concept of a new analytical strategy for in vitro research
Source: Cell Mol Life Sci. 2023 Oct 18;80(11):329. doi: 10.1007/s00018-023-04986-3 (PMC10584734; doi:10.1007/s00018-023-04986-3)
Supplement: Supplementary file 1 — Supplementary file1 (DOCX 2446 KB) [file 18_2023_4986_MOESM1_ESM.docx]

*Supplementary Information*

**Molecular tracking of interactions between progenitor and endothelial cells via Raman and FTIR spectroscopy imaging: a proof of concept of a new analytical strategy for in vitro research**

Karolina Augustyniak^1,2^, Aleksandra Pragnaca^1,2^, Monika Lesniak^3^, Marta Halasa^3,4,8^, Agata Borkowska^3,5^, Ewa Pieta^6^, Wojciech M. Kwiatek^6^, Claudine Kieda^3,7^, Robert Zdanowski^3*^, and Kamilla Malek^1*^

^1^Jagiellonian University in Krakow, Faculty of Chemistry, Department of Chemical Physics, Gronostajowa 2, 30-387 Krakow, Poland

^2^Jagiellonian University in Krakow, Doctoral School of Exact and Natural Sciences,
prof. S. Lojasiewicza 11, 30-348 Krakow, Poland

^3^Military Institute of Medicine - National Research Institute, Laboratory of Molecular Oncology and Innovative Therapies, Szaserow 128, 04-141 Warsaw, Poland

^4^The Houston Methodist Research Institute, Transplant Immunology, Houston, TX, USA

^5^Postgraduate School of Molecular Medicine, Medical University of Warsaw,
Zwirki i Wigury 61, 02-091 Warsaw, Poland

^6^Polish Academy of Science, Institute of Nuclear Physics, Radzikowskiego 152,
31-342 Krakow, Poland

^7^Center for Molecular Biophysics, UPR4301 CNRS, Orleans, France

^8^The Houston Methodist Hospital, Department of Surgery, Houston, TX, USA

**Table S1.** The number of sub-cellular compartments and whole cells detected by Raman and FTIR imaging of endothelial progenitor MAgEC11.5 and brain endothelial MBrMEC cells. RS imaging was performed with a step size of 1 µm.

| *Raman Imaging* | | | | |
| --- | --- | --- | --- | --- |
| *Classes from KMCA* | *MAgEC11.5 (N=42)* | *%* *MAgEC11.5* | *MBrMEC (N=35)* | *%* *MBrMEC* |
| Cytoplasm | 42 | 100% | 35 | 100% |
| Cell nucleus | 42 | 100% | 33 | 94% |
| Perinuclear area | 42 | 100% | 35 | 100% |
| Lipid droplets | 34 | 81% | 22 | 63% |
| *FTIR Imaging* | | | | |
| Whole cells | 40 |  | 48 |  |

**Table S2.** Raman band positions and their assignments to vibrational modes of biomolecules [1–8].

| *Band position [cm^-1^]* | *Assignment to biomolecules and vibrational modes* |
| --- | --- |
| 537 | Proteins; γ(the phenyl rings) |
| 600 | Triacylglycerols; δ(C-O-C) |
| 611 | Cholesterol, cholesterol esters; ν(steroid ring) |
| 647 | Tyr (proteins); δ(C-C) |
| 706 | Cholesterol, cholesterol esters; δ(steroid ring) |
| 729 | A (nucleic acids); ring breathing  Phospholipids; ν_s_(N^+^(CH_3_)_3_) of choline group |
| 755 | Cyt. c and c_1_; ν(porphyrin ring) |
| 770 | U, T, and C (nucleic acids); ring breathing |
| 790 | Nucleic acids; ν_s_(PO_2_) |
| 857 | Tyr (proteins); ν_s_(C-C-N^+^) |
| 867 | Unsaturated fatty acids and triacylglycerols; ν(C-O-O) |
| 884 | Proteins; δ(CH_2_) |
| 1009 | Phe (proteins); ring breathing |
| 1070 | Lipids (*gauche* in acyl backbone); ν(C-C) |
| 1083 | Fatty acids; ν(C-C) |
| 1099 | Phosphate-containing molecules; ν_s_(PO_2_) |
| 1132 | Phospholipids; ν(C-C)  *Cyt. c and c_1_; ν(porphyrin ring) |
| 1175 | Tyr, Phe (proteins); δ(C-H) |
| 1244 | Phosphate-containing molecules; ν_as_(PO_2_) |
| 1251 | Proteins (amide III); ν(C-N), δ(N-H), ν(CH_3_-C) |
| 1261 | Unsaturated lipids; δ(=CH) |
| 1288 | Lipids; δ(CH_2_/CH_3_) |
| 1297 | Fatty acids and triacylglycerols; τ(CH_2_/CH_3_) |
| 1306 | Fatty acids and triacylglycerols; τ(CH_2_/CH_3_) |
| 1318 | G (nucleic acids); ring breathing, δ(C-H)  *Cyt. c and c_1_; δ(C-H) |
| 1340 | Nucleic acids, proteins; ring breathing; δ(C-H)  Lipids; τ/δ(CH_2_/CH_3_) |
| 1381 | A (nucleic acids); δ(CH_3_) |
| 1398 | Nucleic acids; δ(N-H) |
| 1451 | Proteins, lipids; δ(CH_2_, CH_3_) |
| 1585 | A, G (nucleic acids); ring breathing  *Reduced cyt. c, c_1_ and b; ν(methine bridges – C_a_C_m­_,C_a_C_m_H bonds) |
| 1621 | Trp (proteins); ν(C=C) |
| 1656 | α-helices in proteins (amide I); ν(C=O) and δ(N-H) |
| 1660 | Unsaturated fatty acids; ν(C=C) |
| 1670 | Antiparallel β-sheets in proteins; (amide I); ν(C=O) and δ(N-H)  Lipids, fatty acids; ν(C=C) of steroid ring and *trans* configuration |
| 1740 | Triacylglycerols; ν_ester_(C=O) |
| 1749 | Unsaturated triacylglycerols; ν_ester_(C=O) |
| 2853 | Long chain fatty acids; ν_s_(CH_2_) |
| 2879 | Proteins; ν(C-H)-CH_2_ |
| 2895 | Lipids; ν_s_(-C-H)-CH_3_ |
| 2927 | Proteins; ν_as_(CH_3_) |
| 2936 | Lipids; ν(C-H) |
| 2962 | Nucleic acids, lipids; ν_as_(CH_3_), ν_as_CH(-CH_2_) |
| 3013 | Unsaturated fatty acids; ν(=C-H) |

ν – stretching mode, as – asymmetric, s – symmetric; δ – in-plane deformations; γ - out-of-plane deformations;
τ – twisting; cyt – cytochromes; A – adenine; C – cytosine; G – guanine; U – uracil; T – thymine; Tyr – tyrosine; Phe – phenylalanine; Trp - tryptophan; ^*^Bands at 1132, 1318, and 1585 cm^-1^ are assigned to cytochromes only if the ~750 cm^-1^ band is present.

**Table S3.** IR band positions and their assignments to vibrational modes of biomolecules [9–17].

| *Band position [cm^-1^]* | *Assignment to biomolecules and vibrational modes* |
| --- | --- |
| 1050 | DNA; backbone ν(C–O)  Carbohydrates, glycoproteins, glycolipids; ν(C–O)  Cholesterol; ν(C–O) |
| 1067 | Cholesterol esters; ν(CO-O-C) |
| 1085 | Nucleic acids; ν_s_(PO_2_^-^)  Phospholipids; ν_s_(PO_2_^-^) |
| 1108 | Poly/sugars; δ(C-O)  Lactate; δ(COO^-^) |
| 1122 | Ribose (RNA); ν(C-O) |
| 1146 | Poly/sugars; ν(CC-OC) |
| 1166 | Cholesterol esters; ν_as_(CO-O-C) |
| 1238 | Nucleic acids; ν_as_(PO_2_^-^)  Phospholipids; ν_as_(PO_2_^-^) |
| 1307 | Proteins (amide III); ν(C-N) and ν(C-C) |
| 1332 | Phospholipids, fatty acids, triacylglycerols; ν(CH_2_) |
| 1350 | Lipids; δ(C-H) |
| 1388 | Free fatty acids and amino acids; ν_s_(COO^-^) |
| 1396 | Free fatty acids and amino acids; ν_s_(COO^-^) |
| 1458 | Proteins; δ(CH_2_, CH_3_)  C (DNA); δ(NH), ν(CC) |
| 1491 | Proteins; ν(C=C) in aromatic rings |
| 1511 | Tyr (proteins); ν(CC) of the ring  C (methylated DNA); in-plane vibrations of the ring |
| 1545 | Proteins (amide II); δ(N-H) and ν(C-N) |
| 1619 | Intermolecular β-sheets in protein aggregates (amide I); ν(C=O) and δ(N-H) |
| 1645 | α-helices in proteins (amide I); ν(C=O) and δ(N-H) |
| 1666 | 3_10_-helices in proteins (amide I); ν(C=O) and δ(N-H) |
| 1681 | β-turns in proteins (amide I); ν(C=O) and δ(N-H)  G (DNA); ν(C=O) and ν(C=C) |
| 1710 | Fatty acids; ν(C=O) |
| 1725 | Cholesterol esters; ν_ester_(C=O) |
| 1746 | Triacylglycerols; ν_ester_(C=O) |
| 2855 | Long chain FAs; ν_s_(CH_2_) |
| 2879 | Proteins, lipids, nucleic acids; ν_s_(CH_3_) |
| 2895 | Terminal CH_3_ group in acyl chains (lipids); ν(CH) |
| 2912 | Lipids and proteins; ν_as_(CH_2_) |
| 2923 | Lipids and proteins; ν_as_(CH_2_) |
| 2933 | Lipids and proteins; ν_as_(CH_2_) |
| 2965 | Proteins, lipids; ν_as_(CH_3_) |

ν – stretching mode, as – asymmetric, s – symmetric; δ – in-plane deformations; A – adenine, C – cytosine, G – guanine, Tyr – tyrosine.

| Technique | Cluster | Model | Prediction group | RMSEC/R^2^C | RMSEP/R^2^P |
| --- | --- | --- | --- | --- | --- |
| **High-Resolution Raman Imaging**  **24h co-culture** | Cytoplasm | MAgEC11.5=21  MBrMEC=18 | N=22 | 0.09 / 0.992 | 0.16 / 0.975 |
|  | Lipid droplets | MAgEC11.5=17  MBrMEC=11 | N=20 | 0.05 / 0.998 | 0.28 / 0.921 |
|  | Perinuclear area | MAgEC11.5=21  MBrMEC=20 | N=21 | 0.06 / 0.996 | 0.13 / 0.983 |
|  | Cell nucleus | MAgEC11.5=20  MBrMEC=18 | N=20 | 0.14 / 0.980 | 0.20 / 0.963 |
| **FTIR Imaging  24h co-culture** | Single cells | MAgEC11.5=40  MBrMEC=48 | N=68 | 0.13 / 0.980 | 0.16 / 0.973 |
| **FTIR Imaging  4h co-culture** | Single cells | MAgEC11.5=36  MBrMEC=65 | N=65 | 0.13 / 0.983 | 0.17 / 0.971 |

**Table S4.** A summary of data used for models obtained using PLSR together with the determined calibration and prediction errors. RMSEC - Root Mean Square of Error for calibration; R^2^C - R-square of calibration; RMSEP - Root Mean Square of Error for prediction; R^2^P - R-square of prediction.


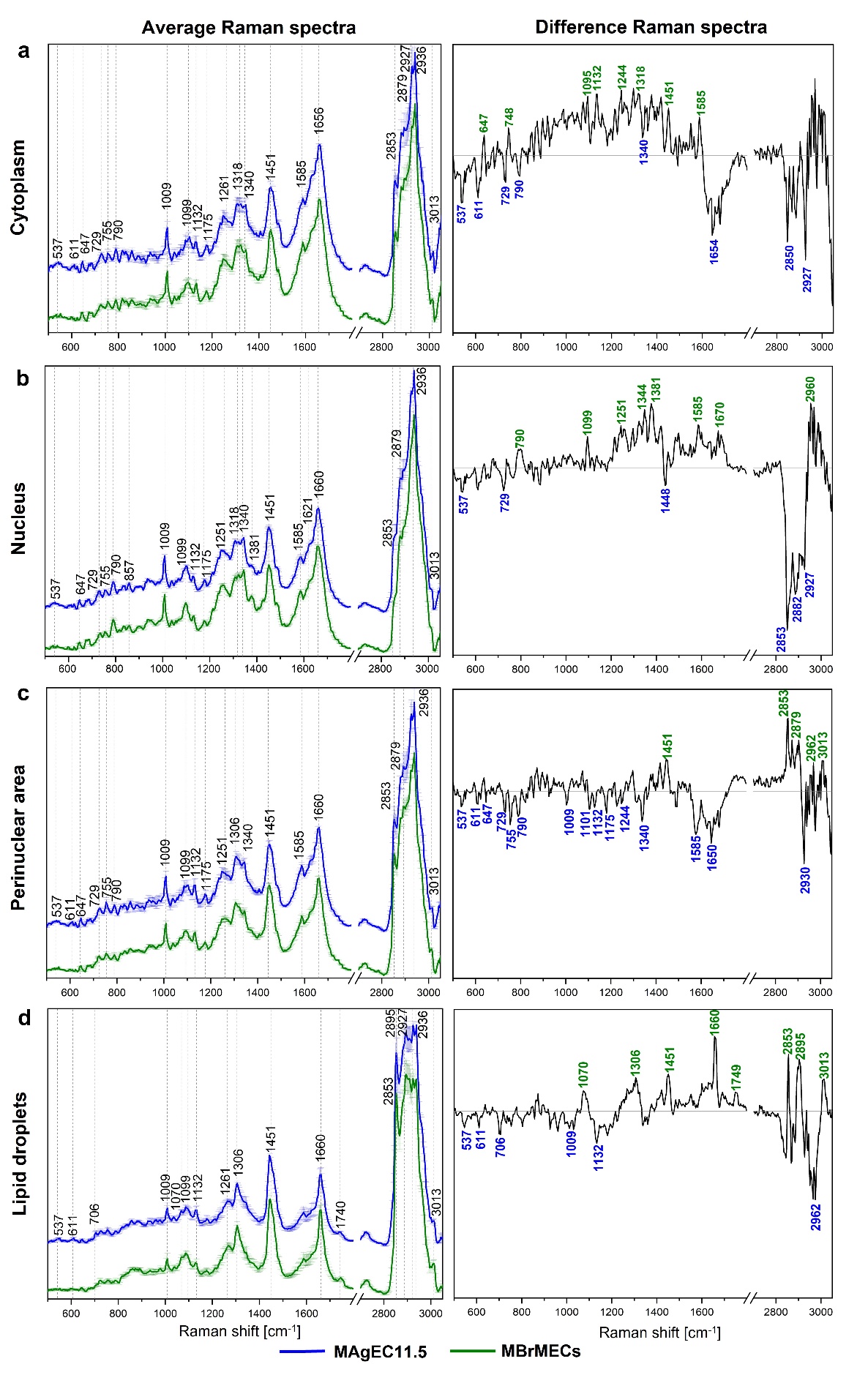


**Fig. S1** Average Raman (*left*) and difference spectra (*right*) of subcellular compartments of endothelial progenitor MAgEC11.5 and brain endothelial MBrMEC cells with marked positions of characteristic Raman bands: (**a**) cytoplasm (N_MBrMEC_ =35, N_MAgEC11.5_=42), (**b**) nucleus (N_MBrMEC_ =33,
N_MAgEC11.5_=42), (**c**) perinuclear area (N_MBrMEC_ =35, N_MAgEC11.5_=42), and (**d**) lipid droplets (N_MBrMEC_ =22, N_MAgEC11.5_=34). Shading denotes ±SD.


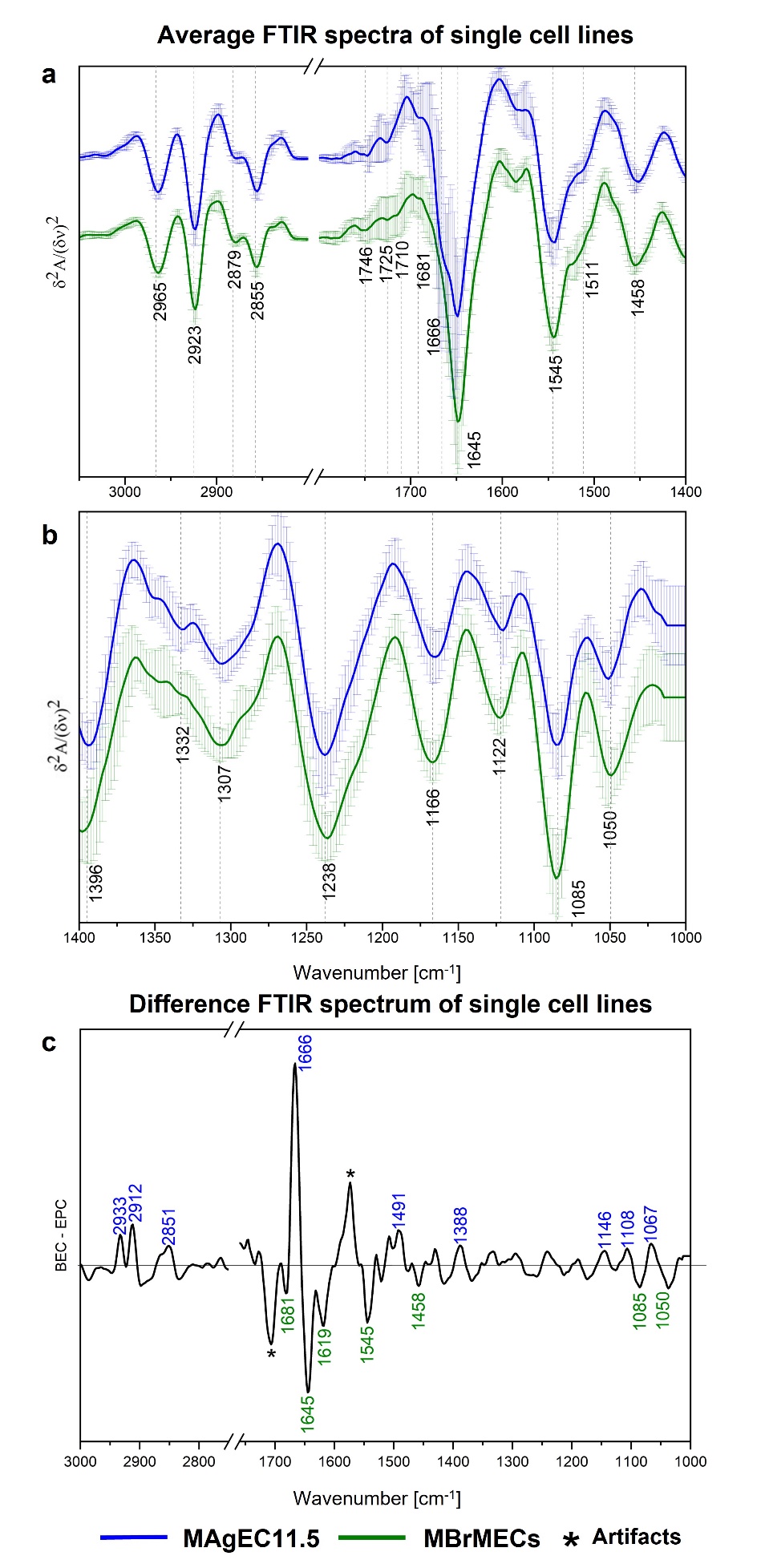


**Fig. S2** Average second derivative FTIR spectra of MAgEC11.5 EPCs (N=40) and MBrMECs (N=48) in the regions of 1400-3050 (**a**) and 1000-1400 cm^-1^ (**b**) and their difference spectrum (**c**) calculated from the subtraction of the EPC from BEC signal. Shading denotes ±SD.


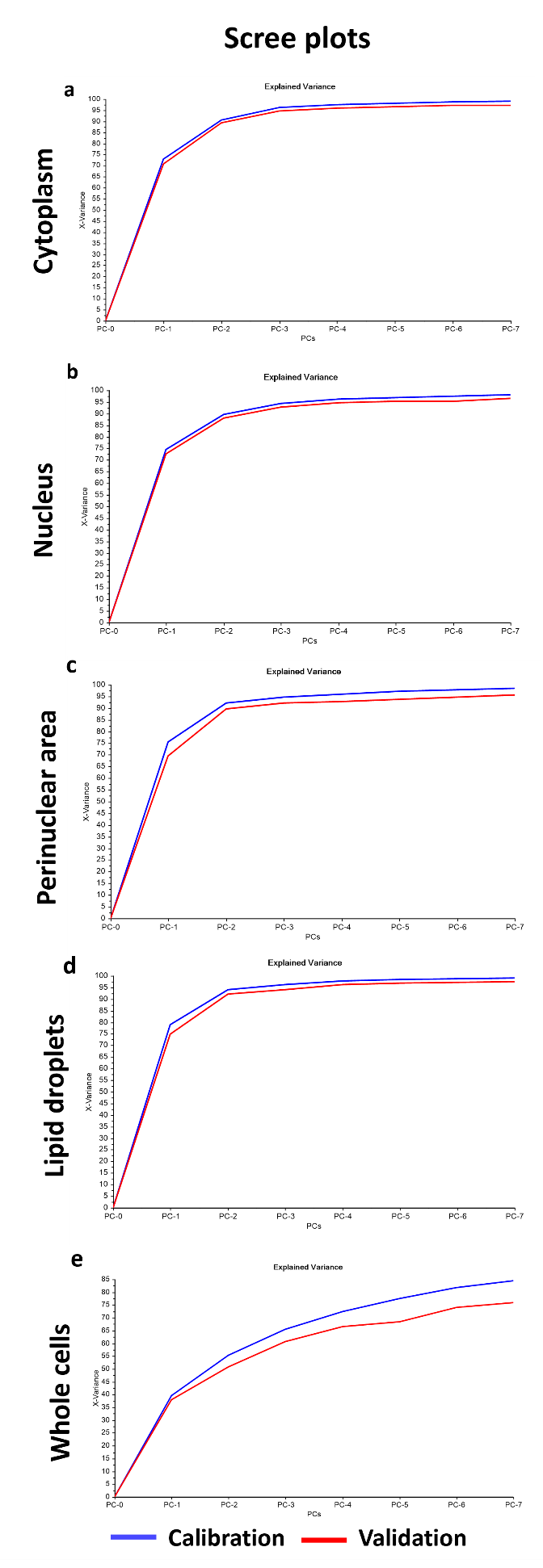


**Fig. S3** The scree plots from Principal Component Analysis performed on Raman spectra of their cellular compartments and FTIR spectra of the whole MAgEC11.5 EPCs and MBrMEC cells: (a) cytoplasm, (b) nucleus, (c) perinuclear area, (d) lipid droplets, (e) whole cells.


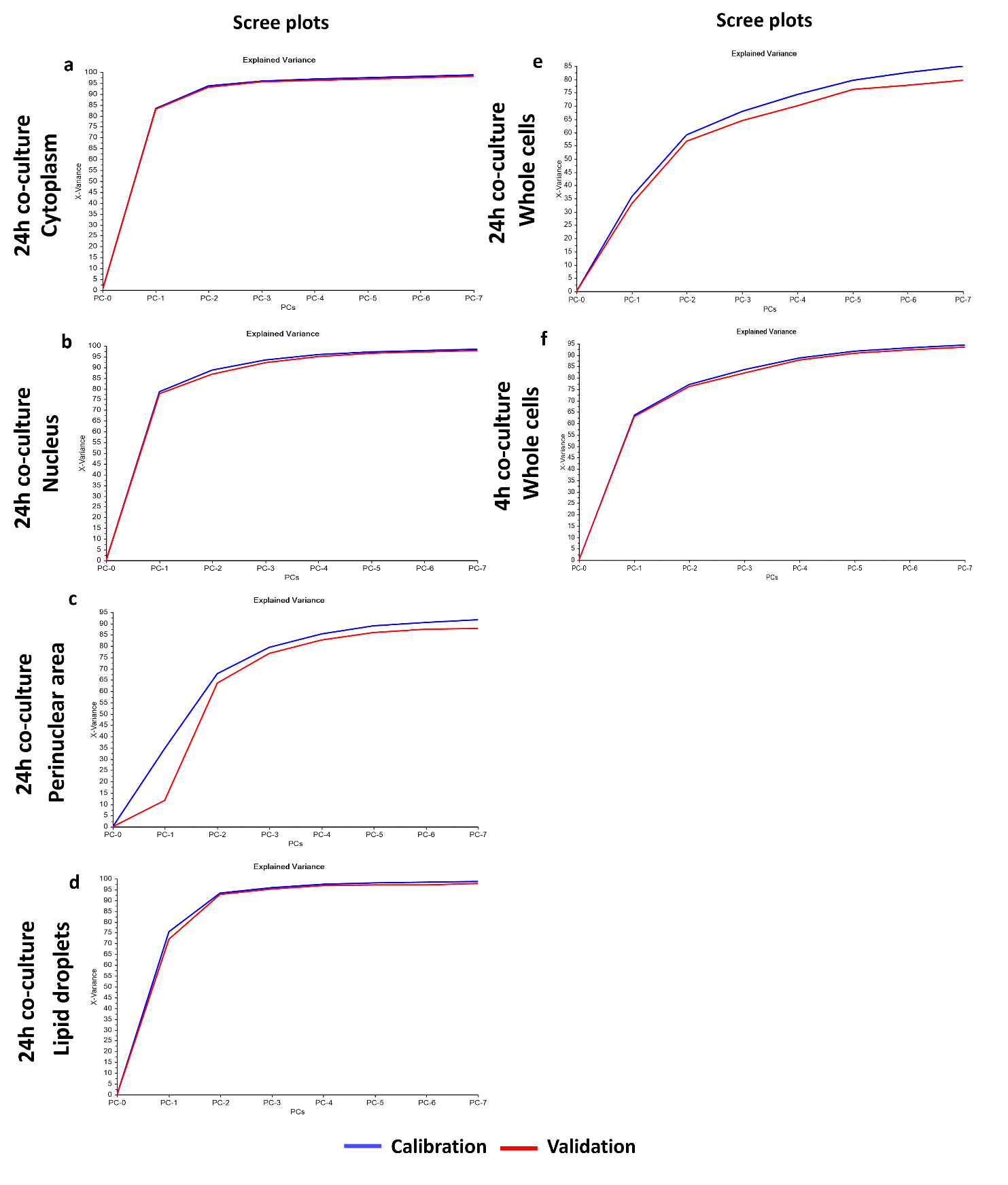


**Fig. S4** The scree plots from Principal Component Analysis performed on Raman spectra of cellular compartments (*left*) and FTIR spectra (*right*) of whole MAgEC11.5 EPCs and MBrMEC cells, and their 24- and 4-hour co-cultured cells.


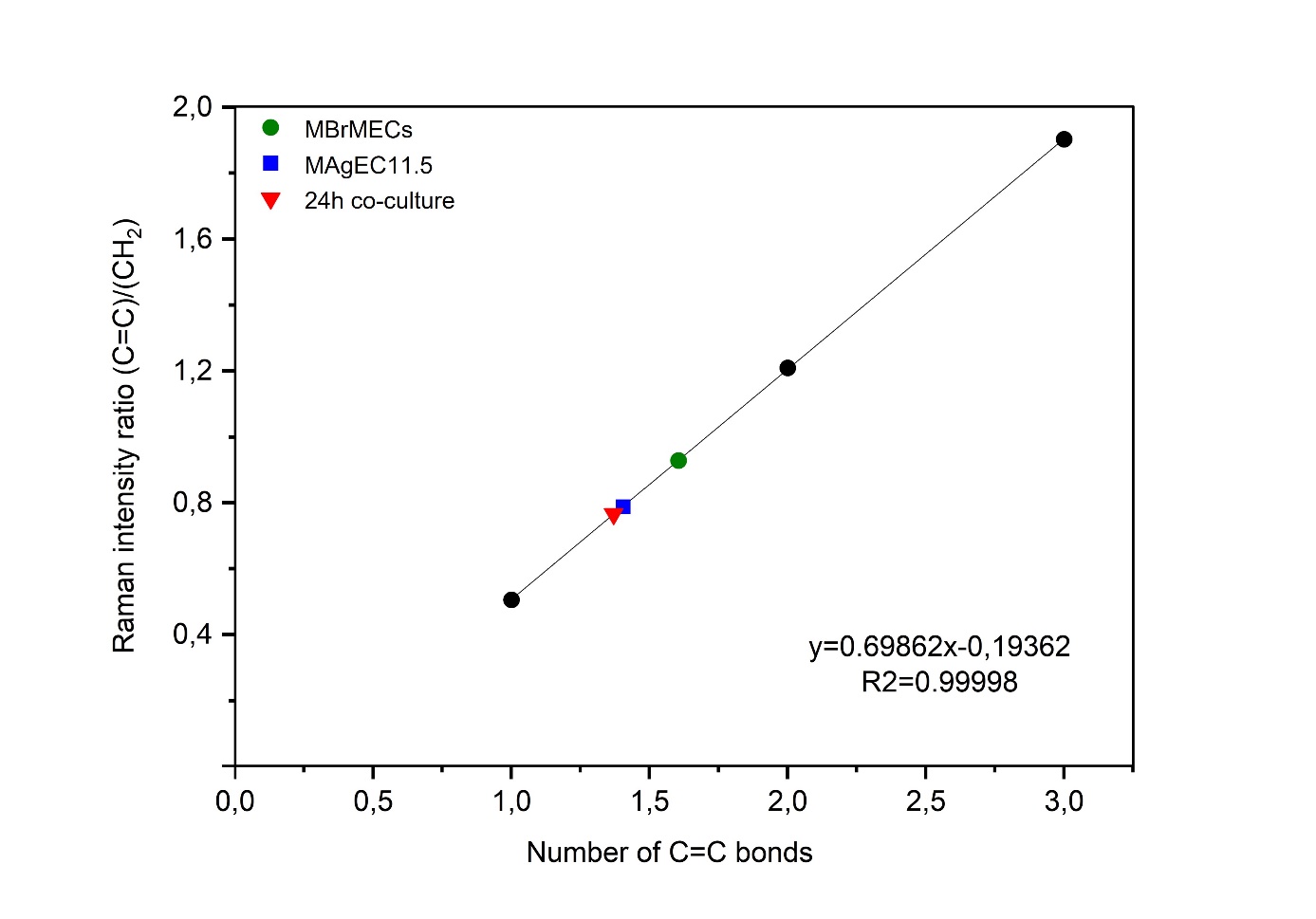


**Fig. S5** A calibration plot of the unsaturation degree of FA acyl chain determined from the Raman spectra of OA – oleic acid (18:1), LA – linoleic acid (18:2), and ALA – α-linolenic acid (18:3) based on the ratio of the 1660 and 1451 cm^-1^ bands. The ratio for MBrMECs, MAgEC11.5, and their 24 h co-cultured cells are marked by a green dot, blue square, and red triangle, respectively.


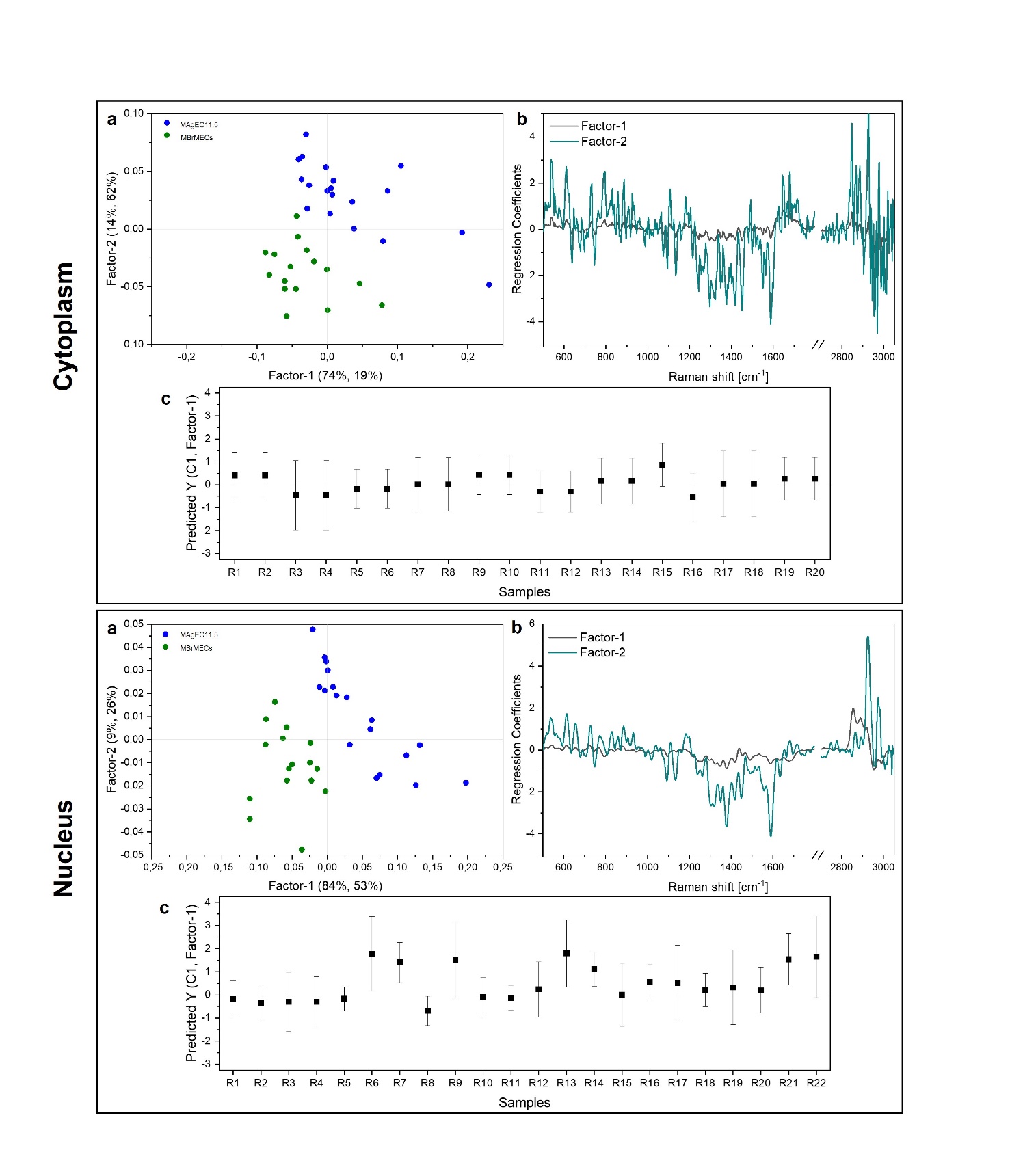


**Fig. S6** PLS regression analysis for the Raman classes of cytoplasm and nucleus. (a) Model built for pure cell lines of MAgEC11.5 EPCs and MBrMECs with (b) plots of the regression coefficient for Factor-1 and Factor-2. (c) Classification of cells from the 24 h co-culture.


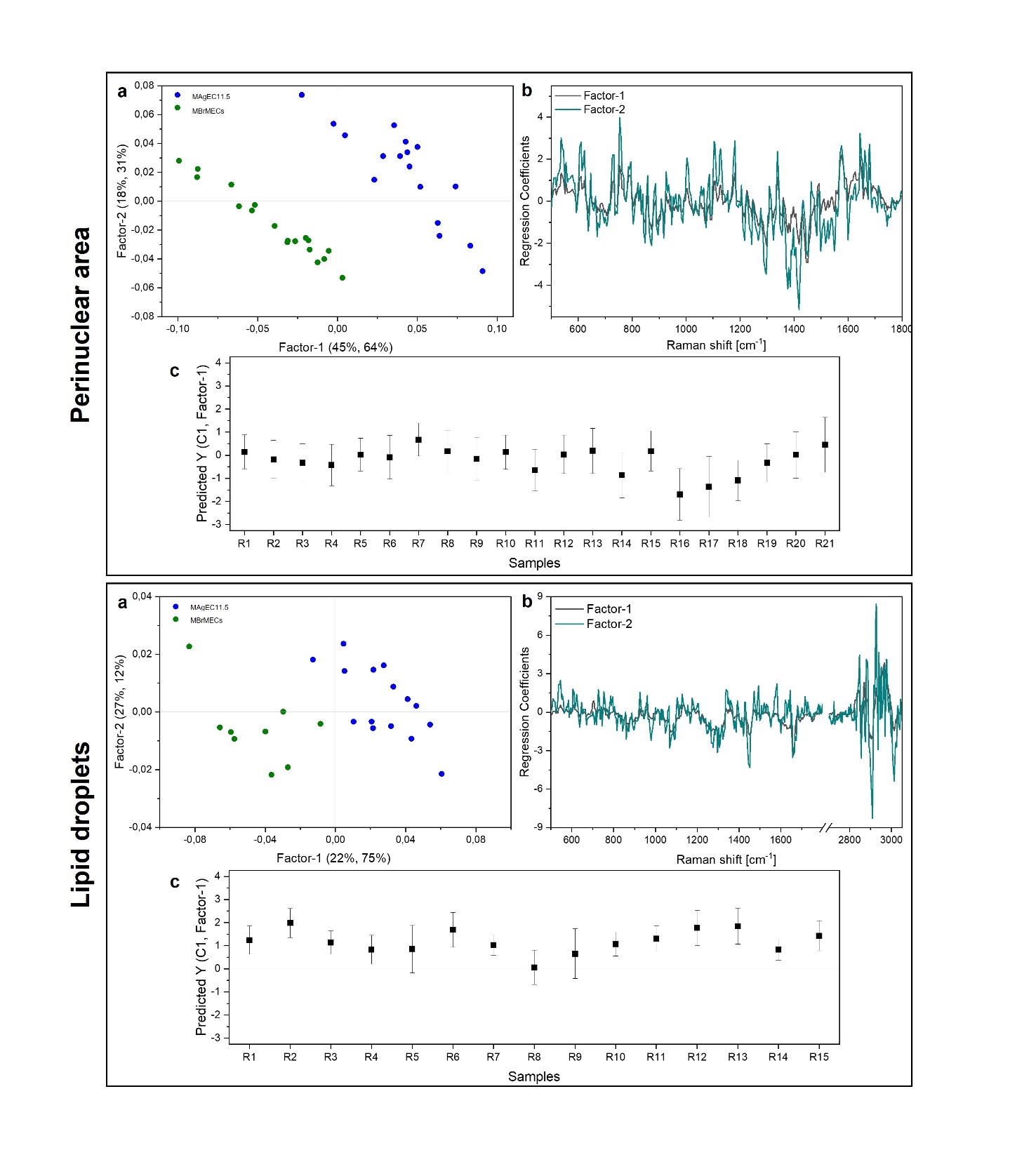


**Fig. S7** PLS regression analysis for the Raman classes of the perinuclear area and LDs. (a) Model built for pure cell lines of MAgEC11.5 EPCs and MBrMECs with (b) plots of the regression coefficient for Factor-1 and Factor-2. (c) Classification of cells from the 24 h co-culture.


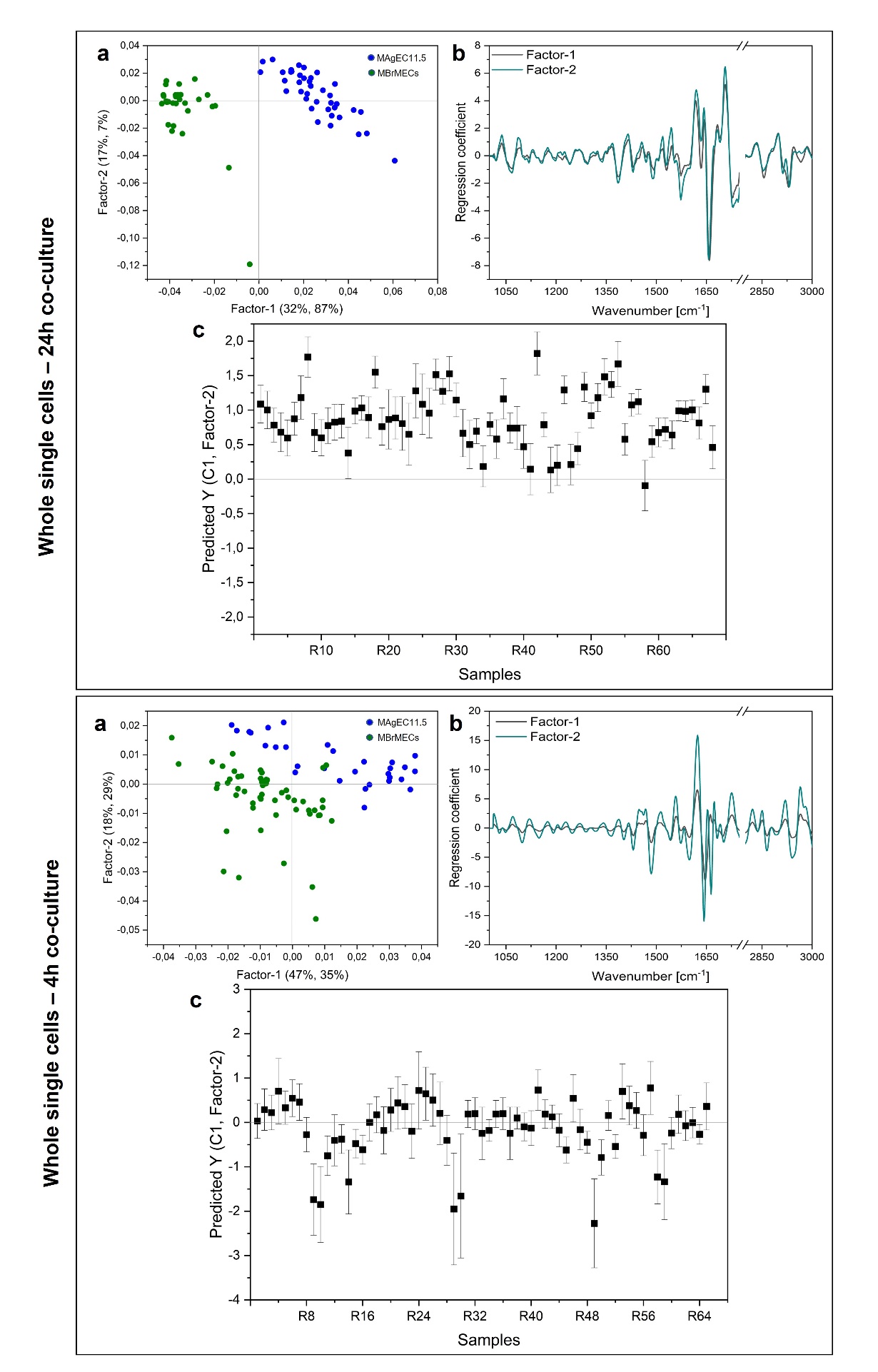


**Fig. S8** PLS regression analysis for IR spectra of the whole cells from 24- and 4 h co-cultures. (a) Model built for pure cell lines of MAgEC11.5 EPCs and MBrMECs with (b) plots of the regression coefficient for Factor-1 and Factor-2. (c) Classification of cells from the co-cultures.

**Bibliography**

1. Bik E, Dorosz A, Mateuszuk L, et al (2020) Fixed versus live endothelial cells: The effect of glutaraldehyde fixation manifested by characteristic bands on the Raman spectra of cells. Spectrochim Acta - Part A Mol Biomol Spectrosc 240:118460. https://doi.org/10.1016/j.saa.2020.118460

2. Majzner K, Chlopicki S, Baranska M (2016) Lipid droplets formation in human endothelial cells in response to polyunsaturated fatty acids and 1-methyl-nicotinamide (MNA); confocal Raman imaging and fluorescence microscopy studies. J Biophotonics 9:396–405. https://doi.org/10.1002/jbio.201500134

3. Czamara K, Majzner K, Pacia MZ, et al (2015) Raman spectroscopy of lipids: A review. J Raman Spectrosc 46:4–20. https://doi.org/10.1002/jrs.4607

4. Prescott B, Steinmetz W, Thomas GJ (1984) Characterization of DNA structures by laser Raman spectroscopy. Biopolymers 23:235–256. https://doi.org/10.1002/bip.360230206

5. Wu M, Pu K, Jiang T, et al (2021) Early label-free analysis of mitochondrial redox states by Raman spectroscopy predicts septic outcomes. J Adv Res 28:209–219. https://doi.org/10.1016/j.jare.2020.06.027

6. Bik E, Mielniczek N, Jarosz M, et al (2019) Tunicamycin induced endoplasmic reticulum changes in endothelial cells investigated: In vitro by confocal Raman imaging. Analyst 144:6561–6569. https://doi.org/10.1039/c9an01456j

7. Matthäus C, Chernenko T, Newmark JA, et al (2007) Label-free detection of mitochondrial distribution in cells by nonresonant Raman microspectroscopy. Biophys J 93:668–673. https://doi.org/10.1529/biophysj.106.102061

8. Meade AD, Clarke C, Draux F, et al (2010) Studies of chemical fixation effects in human cell lines using Raman microspectroscopy. Anal Bioanal Chem 396:1781–1791. https://doi.org/10.1007/s00216-009-3411-7

9. Sahu RK, Argov S, Salman A, et al (2004) Characteristic absorbance of nucleic acids in the Mid-IR region as possible common biomarkers for diagnosis of malignancy. Technol Cancer Res Treat 3:629–638. https://doi.org/10.1177/153303460400300613

10. Szafraniec E, Wiercigroch E, Czamara K, et al (2018) Diversity among endothelial cell lines revealed by Raman and Fourier-transform infrared spectroscopic imaging. Analyst 143:4323–4334. https://doi.org/10.1039/c8an00239h

11. Perez-Guaita D, Kochan K, Martin M, et al (2017) Multimodal vibrational imaging of cells. Vib Spectrosc 91:46–58. https://doi.org/10.1016/j.vibspec.2016.07.017

12. Wiercigroch E, Staniszewska-Slezak E, Szkaradek K, et al (2018) FT-IR Spectroscopic Imaging of Endothelial Cells Response to Tumor Necrosis Factor-α: To Follow Markers of Inflammation Using Standard and High-Magnification Resolution. Anal Chem 90:3727—3736. https://doi.org/10.1021/acs.analchem.7b03089

13. Banyay M, Sarkar M, Gräslund A (2003) A library of IR bands of nucleic acids in solution. Biophys Chem 104:477–488. https://doi.org/10.1016/S0301-4622(03)00035-8

14. Whelan DR, Bambery KR, Heraud P, et al (2011) Monitoring the reversible B to A-like transition of DNA in eukaryotic cells using Fourier transform infrared spectroscopy. Nucleic Acids Res 39:5439–5448. https://doi.org/10.1093/nar/gkr175

15. Staniszewska E, Malek K, Baranska M (2014) Rapid approach to analyze biochemical variation in rat organs by ATR FTIR spectroscopy. Spectrochim Acta - Part A Mol Biomol Spectrosc 118:981–986. https://doi.org/10.1016/j.saa.2013.09.131

16. Molony C, McIntyre J, Maguire A, et al (2018) Label-free discrimination analysis of de-differentiated vascular smooth muscle cells, mesenchymal stem cells and their vascular and osteogenic progeny using vibrational spectroscopy. Biochim Biophys Acta - Mol Cell Res 1865:343–353. https://doi.org/10.1016/j.bbamcr.2017.11.006

17. Lewis RNAH, McElhaney RN (2013) Membrane lipid phase transitions and phase organization studied by Fourier transform infrared spectroscopy. Biochim Biophys Acta - Biomembr 1828:2347–2358. https://doi.org/10.1016/j.bbamem.2012.10.018
